# Clinical outcome and gut development after insulin-like growth factor-1 supplementation to preterm pigs

**DOI:** 10.3389/fped.2022.868911

**Published:** 2022-08-05

**Authors:** Kristine Holgersen, Martin Bo Rasmussen, Galen Carey, Douglas G. Burrin, Thomas Thymann, Per Torp Sangild

**Affiliations:** ^1^Comparative Pediatrics and Nutrition, Department of Veterinary and Animal Sciences, Faculty of Health and Medical Sciences, University of Copenhagen, Frederiksberg, Denmark; ^2^Takeda, Cambridge, MA, United States; ^3^Department of Pediatrics, United States Department of Agriculture/Agricultural Research Service, Children's Nutrition Research Center, Baylor College of Medicine, Houston, TX, United States; ^4^Department of Neonatology, Rigshospitalet, Copenhagen, Denmark; ^5^Department of Pediatrics, Odense University Hospital, Odense, Denmark

**Keywords:** insulin-like growth factor-1, preterm, pig, gut, development

## Abstract

**Background:**

Elevation of circulating insulin-like growth factor-1 (IGF-1) within normal physiological levels may alleviate several morbidities in preterm infants but safety and efficacy remain unclear. We hypothesized that IGF-1 supplementation during the first 1–2 weeks after preterm birth improves clinical outcomes and gut development, using preterm pigs as a model for infants.

**Methods:**

Preterm pigs were given vehicle or recombinant human IGF-1/binding protein-3 (rhIGF-1, 2.25 mg/kg/d) by subcutaneous injections for 8 days (Experiment 1, *n* = 34), or by systemic infusion for 4 days (Experiment 2, *n* = 19), before collection of blood and organs for analyses.

**Results:**

In both experiments, rhIGF-1 treatment increased plasma IGF-1 levels 3-4 fold, reaching the values reported for term suckling piglets. In Experiment 1, rhIGF-1 treatment increased spleen and intestinal weights without affecting clinical outcomes like growth, blood biochemistry (except increased sodium and gamma-glutamyltransferase levels), hematology (e.g., red and white blood cell populations), glucose homeostasis (e.g., basal and glucose-stimulated insulin and glucose levels) or systemic immunity variables (e.g., T cell subsets, neutrophil phagocytosis, LPS stimulation, bacterial translocation to bone marrow). The rhIGF-1 treatment increased gut protein synthesis (+11%, *p* < 0.05) and reduced the combined incidence of all-cause mortality and severe necrotizing enterocolitis (NEC, *p* < 0.05), but had limited effects on intestinal morphology, cell proliferation, cell apoptosis, brush-border enzyme activities, permeability and levels of cytokines (IL-1β, IL-6, IL-8). In Experiment 2, rhIGF-1 treated pigs had reduced blood creatine kinase, creatinine, potassium and aspartate aminotransferase levels, with no effects on organ weights (except increased spleen weight), blood chemistry values, clinical variables or NEC.

**Conclusion:**

Physiological elevation of systemic IGF-1 levels for 8 days after preterm birth increased intestinal weight and protein synthesis, spleen weight and potential overall viability of pigs, without any apparent negative effects on recorded clinical parameters. The results add further preclinical support for safety and efficacy of supplemental IGF-1 to hospitalized very preterm infants.

## Introduction

About 10% of all infants are born preterm [<37 weeks gestation, (1)]. In preterm infants, nutrient metabolism, immunity and many organ systems are immature which complicates their adaptation to extrauterine life. Birth is associated with a rapid fall in circulating insulin-like growth factor-1 (IGF-1) levels due to the abrupt loss of fetal or maternal IGF-1 supply. After preterm birth, the subsequent postnatal increase in IGF-1 levels occurs slowly (2). Especially very preterm infants (<32 weeks gestation) show an extended period of low postnatal IGF-1 levels with a more than 50% reduction in serum IGF-1 levels relative to term infants and gestational age-matched fetuses in utero (2–4). Low levels of circulating IGF-1 have been associated with several complications of prematurity (5), including growth restriction (6–9), systemic inflammation [early- or late-onset sepsis, (10, 11)], lung complications [bronchopulmonary dysplasia, BPD, (12)], gut inflammation [necrotizing enterocolitis, NEC, (9)], eye problems [retinopathy of prematurity, ROP, (9, 13)] and brain damage [intraventricular hemorrhage, IVH, (14, 15)]. One or more of these major neonatal morbidities occur in >60% of infants born extremely preterm [<28 weeks gestation, (16, 17)]. In a global phase 2 clinical study, supplemental recombinant human (rh) IGF-1/rhIGF binding protein-3 (rhIGF-1) reduced BPD and IVH severity in extremely preterm infants (18). A large multi-center international trial is now ongoing to verify if supplemental IGF-1, within normal physiological levels, will prevent later morbidities in extremely preterm infants (ClinicalTrials.gov registry NCT03253263).

IGF-1 is a mitogenic polypeptide with specific receptors in many tissues and organs, considered essential for growth and development during pregnancy, infancy and childhood (6–8, 19–21). Fetal and maternal IGF-1 blood levels increase with gestational age, indicating a significant role of IGF-1 in fetal development, particularly in the third trimester (4, 22, 23). In fetuses and neonates, insulin stimulates hepatic IGF-1 production and release into the bloodstream (4, 24, 25). The major part of circulating IGF-1 in children and adults is bound to IGF-binding protein (IGFBP)-3 and an acid labile subunit (ALS) that increase the half-life and regulate bioavailability (26). In addition to the liver, many tissues synthesize IGF-1 locally, facilitating paracrine and autocrine actions of IGF-1 in addition to endocrine functions (27, 28).

Colostrum contains high concentrations of IGF-1 (29, 30). The level declines rapidly during the first few days of lactation and the higher mitogenic activity of colostrum compared with mature milk may partly be attributed to the growth-promoting effects of IGF-1 (31). IGF-1 has a local effect on proliferation and maturation of the gut mucosa but is not normally absorbed intact into the circulation. Colostrum feeding reduces the incidence and severity of NEC-like lesions in preterm pigs compared to formula feeding (32–34), while studies in neonatal piglets have observed increased growth, epithelial absorption and enzyme activity in the small intestine after enteral IGF-1 supplementation (35–37). Furthermore, parenteral IGF-1 administration attenuates artificially induced intestinal damage in weaned rodents by decreasing apoptosis and increasing proliferation of epithelial cells (38–40). Preterm pigs show reduced circulating IGF-1 levels compared with term pigs (41) and our previous studies show that two daily subcutaneous injections of rhIGF-1 (1.0 mg/kg/d) reduce the severity of NEC and increase intestinal brush border peptidase activities and villus heights (42). In these studies, treatment also increased circulating neutrophil counts, thereby partly preventing postnatal neutropenia (42–44), and increased circulating IGF-1 to mean levels of ~40 ng/mL, still well below values in term suckling pigs [~70 ng/mL, (42)]. Clinical safety and efficacy of rhIGF-1 given at higher doses, near the physiological levels of term pigs, and for a longer period are not known.

IGF-1 exerts its receptor-mediated action in various organs supporting cell survival and growth, with or without effects on nutrient metabolism and glucose homeostasis. IGF-1 has insulin-like activity and increases cellular glucose uptake and utilization in the developing gut (37) and brain (45). Accordingly, IGF-1-deficient young mice show hyperinsulinemia and peripheral insulin resistance (46, 47). Clinical investigations indicate that IGF-1 may protect against development of glucose intolerance or type-2 diabetes (48) and subcutaneous IGF-1 treatment reduces insulin required to maintain normoglycemia in type-1 diabetes patients (49–51). Dysregulated glucose metabolism is a common complication of premature birth. Initially, preterm infants may suffer from hypoglycemia, which is partly related to their low glycogen stores and impaired glucogenolytic/gluconeogenic activity (52–54). Continuous parenteral glucose infusion is often required to maintain glucose levels within the first week after preterm birth and, especially in preterm pigs, periods of hypoglycemia may prevail for several weeks if fed exclusive enteral nutrition (41). Conversely, hyperglycemia (blood glucose ≥10.0 mmol/L) is also a common observation during the first postnatal weeks due to a combination of insulin resistance, pancreatic β-cell immaturity and parenteral nutrient infusion (54, 55). The risk of hyperglycemia is dependent on gestational age and birth weight and associated with increased mortality and morbidities [e.g., neurodevelopmental delay, IVH and sepsis, (54–56)]. Low serum IGF-1 levels are associated with hyperglycemia just after birth in very low birth weight infants (9). Reduced mortality, especially from sepsis, is observed after insulin treatment in critically ill adults (57) and extremely preterm infants (55) with hyperglycemia. Conversely, early insulin therapy of preterm infants without severe hyperglycemia may be harmful (58).

As a follow-up to our previous study (42), the objective of this study was to further evaluate preclinical safety and efficacy of physiological increases in circulating IGF-1 levels during the first weeks after preterm birth, when the most critical postnatal adaptation occurs. Mode and length of IGF-1 administration are crucial for clinical feasibility. Hence, we tested the effects of both longer-term, intermittent and shorter-term, continuous dosing on gut, metabolism and additional immune–related outcomes. Using the preterm pig as a model of preterm infants, we hypothesize that restoring circulating IGF-1 levels close to those in term counterparts will improve clinical and paraclinical outcomes, gut development and glucose homeostasis. To test our hypothesis, rhIGF-1 was first administered *via* subcutaneous (s.c.) catheters three times daily from day 1–9 after preterm birth (Experiment 1). An additional study tested the effects of a shorter protocol where rhIGF-1 was administrated continuously *via* parenteral nutrition from day 1–5 (Experiment 2).

## Materials and methods

Animal studies were conducted in accordance with the European Communities Council Directive 2010/63/EU for the protection of animals used for experimental purposes and approved by the Danish Animal Experiments Inspectorate, Ministry of Environment and Food of Denmark. All procedures followed the ARRIVE guidelines for animal experimentation (59) and personnel participating in the studies was blinded to the treatments.

### Delivery, housing and surgical procedures

**Figure 1:**
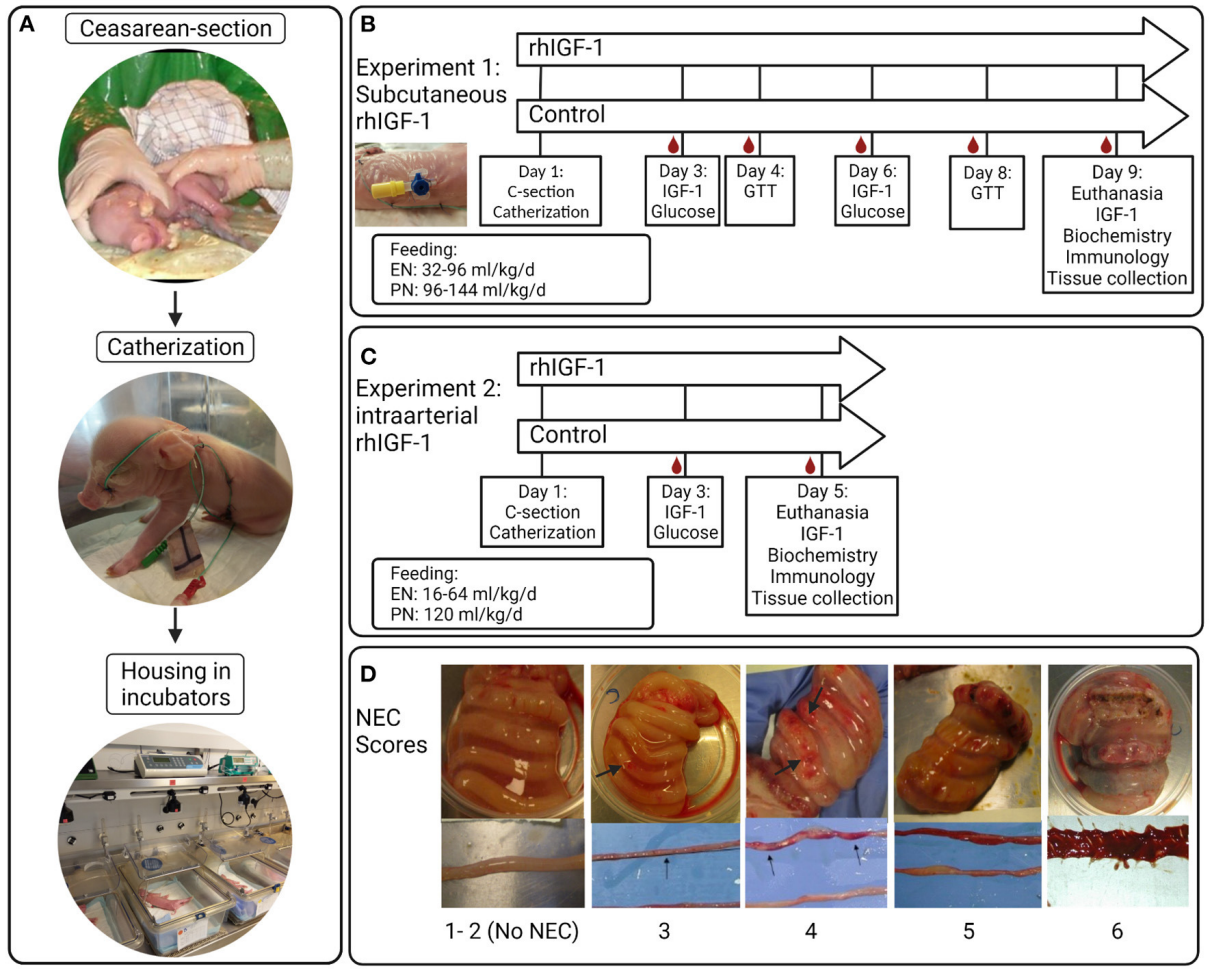
Overview of study procedures, experimental design and NEC scores for small intestine and colon. **(A)** Preterm pigs are delivered by cesarean section, resuscitated and fitted with an orogastric feeding tube and umbilical artery catheter. Pigs are supplemented parenteral nutrition to meet energy requirements and housed individually in heated incubators. **(B)** Blood sampling, injections and feeding regimens in Experiment 1 with subcutaneous rhIGF-1 administration. **(C)** Blood sampling, injections and feeding regimens in Experiment 2 with intra-arterial rhIGF-1 administration. **(D)** Examples of small intestinal and colonic tissues with NEC scores 1-6; 1 = absence of lesions, 2 = local hyperaemia, 3 = hyperaemia, extensive edema and local hemorrhage, 4 = extensive hemorrhage, 5 = local necrosis or pneumatosis intestinalis, 6 = extensive necrosis and pneumatosis intestinalis. Note the hemorrhagic lesions (arrows). rhIGF-1, recombinant human insulin-like growth factor-1; IGF-1, insulin-like growth factor-1; GTT, glucose tolerance test; NEC, necrotizing enterocolitis. The figure was created using BioRender.

The piglets (Landrace x Yorkshire x Duroc) were delivered by cesarean section at 90% of expected gestation (full term = 117 ± 2 days), housed in heated incubators and infused with parental nutrition (PN) and enteral nutrition (EN) *via* umbilical and orogastric catheters, respectively, as previously described (42) (Figure 1A). The EN was composed of commercially available products used for feeding infants. Formula recipe, PN and EN composition, and total nutrient supply, are shown in Supplemental Tables 1–3. Autopsy was performed on all randomized animals. However, only pigs surviving at least until day 5 in Experiment 1 and until day 4 in Experiment 2 were included in organ sampling and further laboratory analyses.

### Experiment 1: Longer-term subcutaneous rhIGF-1 administration

A 1:1 molar ratio of the non–covalent complex rhIGF-1/rhIGFBP-3 (rhIGF-1, mecasermin rinfabate) and formulation vehicle were provided by Takeda, Lexington, MA, United States. Based on literature studies in infants and pigs, we aimed for a circulating IGF-1 range of 30–110 ng/ml (3, 4, 41, 42). 73 piglets were delivered from three sows. Five piglets were euthanized before randomization due to multi-organ immature dysfunctions (mainly respiratory distress). Within each litter, pigs were block-randomized according to birth weight and gender into two groups: Pigs given 0.75 mg/kg rhIGF-1 (*n* = 34, male/female: *n* = 16/18) or equivalent volumes of vehicle (*n* = 34, male/female: *n* = 17/17) three times daily at 6.30 am, 2.30 pm and 10.30 pm *via* the s.c. catheter until they were euthanized on day 9. The experimental setup and placement of s.c. catheters are shown in Figure 1B. The predetermined volume of PN on individual study days (up to 144 mL/kg/d, Supplemental Table 2) was adjusted for individual pigs according to any clinical signs of dehydration, diarrhea or edema. Increasing amounts of enteral formula was provided from day 1 (32–96 mL/kg/d). Glucose concentration in the PN bags was kept at a relatively high level (140–180 g/L, Supplemental Table 1), resulting in a total supply of carbohydrate of ~10–25 g/kg/d across the study days from PN and EN (Supplemental Table 2). The purpose of the relatively high PN glucose supply was to challenge the glucose metabolic capacity of the pigs, thereby better allowing rhIGF-1 treatment to show effect on glucose clearance and insulin sensitivity.

### Experiment 2: Short-term continuous systemic rhIGF-1 administration

Two sows delivered 45 piglets. Seven pigs were euthanized before randomization due to multi-organ immature dysfunctions (mainly respiratory distress). The IGF-1 product and target treatment goals were identical to those in Experiment 1. Within each litter, pigs were block-randomized according to birth weight and gender into two groups: Pigs given continuous administration of 2.25 mg/kg/day rhIGF-1 (*n* = 19, male/female: *n* = 11/8) or equivalent volumes of vehicle (*n* = 19, male/female: *n* = 11/8) *via* the umbilical arterial catheter, together with the PN solution, until they were euthanized on day 5. The experimental setup is shown in Figure 1C. Supply of PN was kept at 120 mL/kg/d and EN increased from day 1–5 (16–80 mL/kg/d), resulting in a carbohydrate supply of 11–13 g/kg/d (Supplemental Table 3).

### Clinical observations and *in vivo* tests

Pig health status was evaluated daily by body weight along with clinical and fecal status twice a day using a validated scoring system (41) (except that fecal score 5 included diarrhea with visual blood). Pigs were euthanized if severe respiratory distress, sepsis, severe NEC symptoms or other severe clinical symptoms occurred after start of treatment but before the end of the experiments (denoted “all-cause mortality”). Pigs dying earlier than 12 h postnatally (on day 1) were excluded from all-cause mortality analysis. Intestinal permeability was assessed by the lactulose-mannitol method, just prior to euthanasia, as previously described (42).

### Circulating IGF-1 levels

In Experiment 1, blood samples were taken *via* the umbilical catheter three (on day 6), five (on day 3) and eight (on day 9) hours after injection and *via* intracardial puncture under anesthesia just before euthanasia. In Experiment 2, blood samples were obtained on day 3 and 5 *via* the umbilical catheter, and *via* intracardial puncture under anesthesia just before euthanasia. Comparison between IGF-1 levels in samples obtained from the umbilical catheters and intracardial puncture indicated IGF-1 contamination *via* the umbilical catheter, resulting in falsely elevated IGF-1 measurements (Supplemental Table 4). Thus, in Experiment 2, only day 5 samples obtained *via* intracardial puncture were used to quantify circulating IGF-1 levels. Blood was collected in EDTA-coated tubes and plasma was separated by centrifugation at 2,000 x g for 10 min and stored at −80°C. All samples were analyzed using a human IGF-1 ELISA kit (Mediagnost GmbH, Reutlingen, Germany) in a bioanalytical testing laboratory (PPD Laboratories, Richmond, Virginia). The lower limit of quantitative detection was 20 ng/mL, but values at 10–20 ng/mL were included in the analyses to estimate low values in control animals. Values lower than 10 ng/mL were assigned a value of 5 ng/mL in the quantitative evaluations.

### Blood biochemistry, hematology and systemic immune analyses

Blood was collected on the day of euthanasia in heparin vacutainers (BD Diagnostics, Oxford, United Kingdom) *via* the umbilical catheter or jugular vein. Biochemistry of plasma and cell counting of whole blood were analyzed using an Advia 1,800 Chemistry System and an Advia 2120i Hematology system (Siemens Healthcare, Ballerup, Denmark), respectively.

For T cell subset characterization, FACS lysing solution (BD Biosciences, Franklin Lakes, New Jersey), was added to 100 μL blood to lyse the red blood cell and the samples were washed twice in phosphate-buffered saline (PBS) containing 1% fetal bovine serum (FBS). The leukocytes were incubated with fixation/permeabilization buffer (Invitrogen, Carlsbad, California) for 30 min at 4°C in the dark, washed with permeabilization buffer [10% permeabilization concentrate (Invitrogen) in distilled water] and blocked with porcine serum (Thermo Fisher, Roskilde, Denmark) for 15 min 4°C in the dark. Subsequently, the leucocytes were stained with a mastermix containing the following monoclonal antibodies (mAbs); PE-Cy7- conjugated mouse anti-pig CD3ε (clone BB23–8E6–8C8, BD bioscience), FITC-conjugated mouse anti-pig CD4α (clone MIL17, Bio-Rad Laboratories, Hercules, California), PE-conjugated mouse anti-pig wCD8α (clone MIL12, Bio-Rad Laboratories) and APC-conjugated rat anti-mouse FOXP3 Monoclonal Antibody (clone FJK-16s, Thermo Fisher Scientific). The stained cells were washed twice in permeabilization buffer and examined using a multicolor BD Accuri C6 flow cytometer (BD Biosciences) equipped with BD FACSDiva software. The lymphocytes population was gated based on the FSC vs. SSC plot located FSClowSSClow and 10.000 CD3 + events were analyzed in each sample. T cells, T- helper cells (Th), cytotoxic T cells (Tc) and regulatory T cells (Tregs) were identified as CD3 + lymphocytes, CD3 + CD4 + CD8- lymphocytes, CD3 + CD4-CD8 + lymphocytes, and CD3 + CD4 + FoxP3 + lymphocytes, respectively.

The phagocytic activity of neutrophils against gram-negative bacteria was assessed using pHrodo Red *E. coli* (560/585 nm) BioParticles Phagocytosis Kits for flow cytometry (Thermo Fisher Scientific, Roskilde, Denmark). Samples were prepared as previously described (43) and analyzed on the flow cytometer. The neutrophil population was gated based on the FSC vs. SSC plot located FSChighSSChigh and 5,000 neutrophils were analyzed in each sample. The proportion of pHrodo + neutrophils in the neutrophil population indicated the phagocytic activity (%) and the median fluorescence intensity (MFI) represented the phagocytic capacity of the neutrophils.

To characterize the pigs' ability to regulate systemic inflammatory response, 294 μL whole blood was incubated with a TLR4 agonist (lipopolysaccharide, LPS, *E.coli* O127:B8, Sigma Aldrich, Søborg, Denmark) for 5 h at 37°C, 5% CO_2_. After incubation, the blood was centrifuged at 2,000 × g, 4°C for 10 min and supernatants were collected for analysis of TNF-α by ELISA (R&D Systems, Abingdon, UK), following the manufacturer's guidelines. The plasma samples were diluted 1:2.5. The lowest standard of the analyses was 31.3 pg/mL and measurements less than this were assigned a value of 15.8 pg/mL. The TNF-α response to LPS stimulation was analyzed as the percentage change in TNF-α levels in LPS-stimulated vs. unstimulated samples for each animal.

### Glucose tolerance test, insulin and glucose levels

In Experiment 1, subgroups of pigs underwent an intra-arterial (i.a.) glucose tolerance test (IAGTT) on day 4 (*n* = 11/group) and day 8 (*n* = 6–7/group). After a 4 h fasting period, an i.a. bolus of 1.0 mg/kg glucose (from a 50% glucose solution) was administrated and the catheter was flushed thoroughly with saline. Then arterial blood was sampled in EDTA tubes at 0, 5, 10, 20, 40, and 60 min. Samples were centrifuged at 2,000 x g for 10 min and plasma was collected and stored at −80°C. Samples for basal insulin were obtained on day 3 and 6 in Experiment 1, and on day 3 and 5 in Experiment 2. Plasma insulin was analyzed by a commercial porcine ELISA kit (Mercodia, Salem, NC). Measurements below level of detection (2 mU/L) were assigned to 2 mU/L. Plasma glucose responses from the IAGTT was measured using a colorimetric assay (Thermo Scientific Waltham, MA), whereas basal glucose levels were measured by a glucometer (ACCU-CHEK, Roche Diagnostics, Hvidovre, Denmark) following ear vein puncture on day 3, 4, 6, and 8 in Experiment 1 and on day 3 in Experiment 2.

### Tissue collection and NEC evaluation

Pigs were euthanized by intracardial administration of sodium-pentobarbital (Euthanimal, Scanvet, Denmark) on day 9 and 5, in Experiment 1 and 2, respectively. The stomach, proximal, middle and distal small intestine and colon were evaluated for macroscopic signs of NEC by two independent observers, as previously described (42, 60). NEC scores are shown in Figure 1D. “NEC” was defined as a score of at least 3 in one or more gut regions, whereas a score of at least 4 in one or more gut regions was defined as “severe NEC”. An average NEC severity score of the total gastrointestinal tract was calculated as the mean of the highest score of the small intestinal (proximal, middle or distal), colon and stomach regions. Pigs dying earlier than 12 h postnatally (on day 1) were excluded from NEC analysis. Organs were weighed and tissue from small intestine and colon was snap frozen and stored at −80°C to evaluate enzyme activities and levels of pro-inflammatory cytokines.

### Gut protein synthesis, morphology, enzyme activity and inflammatory markers

Fractional rates of protein synthesis were measured in pigs euthanized day 8–9 in Experiment 1 and day 5 in Experiment 2, with a flooding dose (10 ml/kg) of L-phenylalanine (Phe) (1.50 mmol/kg) containing L-[ring-2H5]Phe at 30 mol % (0.45 mmol/kg) (Cambridge Isotope Laboratories, Tewksbury, MA) injected i.a. *via* the umbilical catheter 30 min prior to euthanasia. Collected distal small intestine samples were snap-frozen in liquid nitrogen and stored at −80°C. Analysis for tracer enrichment in the intestine tissue and calculation of the fractional protein synthesis rate (FSR, % protein mass synthesized/day) were performed as previously described (61). Villus height and crypt depth in the proximal small intestine were measured in paraformaldehyde-fixed slices following H&E and Alcian-blue/periodic acid-schiff staining (42). In Experiment 1, the degree of proliferation was measured with Ki67 immunohistochemical (IHC) staining in eight representative pictures of proximal small intestine sections and reported as the mean Ki67 positive area of the total nuclei area in tunica mucosa (%), as previously described (42). The degree of apoptosis was assessed by caspase-3 IHC staining and measured as mean caspase-3 positive area relative to the total mucosal cell area (%) in eight representative pictures. Brush border enzyme activities were measured in the proximal and distal small intestine in both experiments. Assays were performed using specific substrates, as described previously (62). Interleukin (IL)-1β, −6, and −8 levels in colon homogenates were analyzed using DuoSet ELISA kits (R&D systems, Minneapolis, MN) following the manufacturer's instruction. Each sample was tested in duplicate.

### Statistical analysis

The statistical software package R (version 3.6.1) was used for statistical analyses. Statistical significance of difference in continuous variables was analyzed by linear models, if data were normally distributed. The model was tested for normality, data was log-transformed when required and non-parametric analysis was applied if data could not be transformed to reach normal distribution. Binary data were analyzed by a logistic regression model. Ordered categorical outcomes (e.g., NEC score) were analyzed by a proportional odds logistic regression model. Repeated measurements were analyzed by the linear mixed effect model. The above mentioned models were adjusted for possible confounders like litter, sex and birth weight. A *p* value < 0.05 was considered statistically significant and 0.05 ≤ *p* < 0.10 was noted and discussed as a tendency to an effect. Values were expressed as mean ± standard error of mean (SEM), unless otherwise specified. Levels of significance were assigned as ^*^p < 0.05, ^**^p < 0.01, ^***^p < 0.001.

## Results

### Experiment 1: Longer-term subcutaneous rhIGF-1 administration

#### Circulating IGF-1 levels

**Figure 2:**
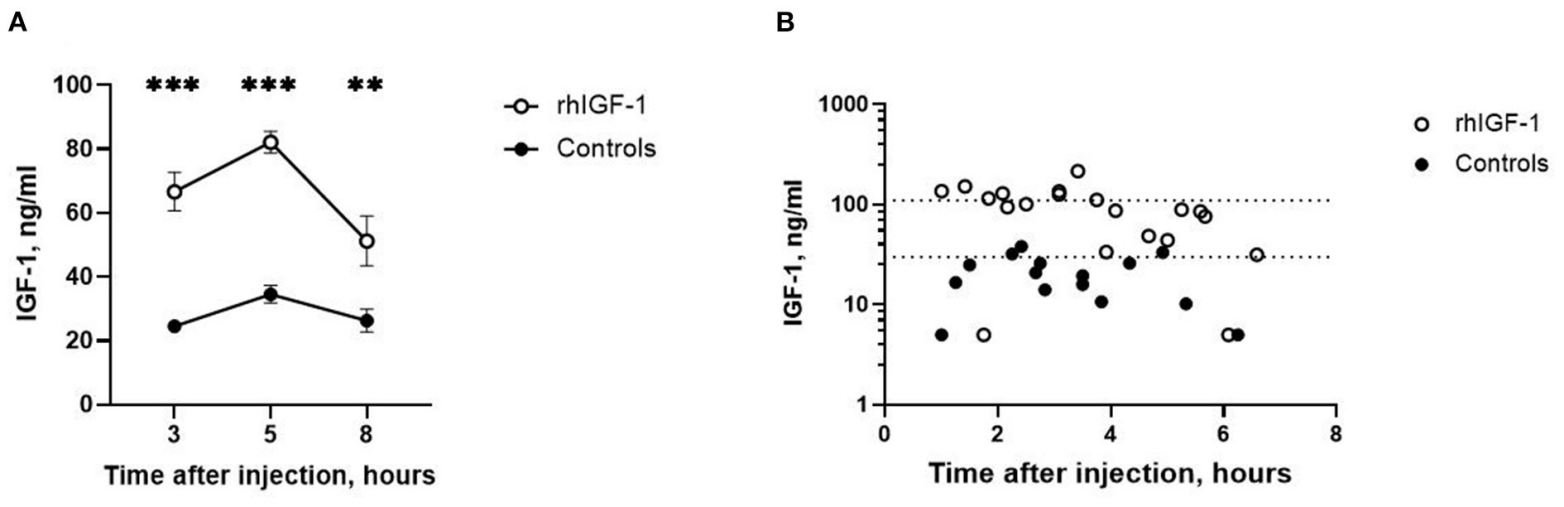
Circulating IGF-1 levels in Experiment 1. **(A)** Plasma IGF-1 levels in rhIGF-1 and control preterm pigs after a subcutaneous injection of rhIGF-1 or vehicle (mean ± SEM, *n* = 13–26). **(B)** Plasma IGF-1 levels at euthanasia 1–7 h after the last subcutaneous injection of rhIGF-1 or vehicle on day 9. Each point represent one pig. The dotted lines indicate the desired physiological range. IGF-1, insulin-like growth factor-1; rhIGF-1, recombinant human insulin-like growth factor-1. **p < 0.01, ***p < 0.001.

Three, five and eight h after s.c. rhIGF-1 bolus, the plasma IGF-1 levels in rhIGF-1 pigs were 66.6 ± 6.0 ng/mL (*n* = 20), 82.1 ± 3.4 ng/mL (*n* = 25) and 51.2 ± 7.8 ng/mL (*n* = 12), respectively (mean ± SEM, Figure 2A). One rhIGF-1 pig was excluded from all analyses because of a malfunctioning s.c. catheter, indicated by repeatedly low IGF-1 plasma levels despite the rhIGF-1 injections (Supplemental Table 5). In corresponding control pigs, the plasma IGF-1 levels were 24.6 ± 1.8 ng/mL (*n* = 23), 34.6 ± 2.7 ng/mL (*n* = 25) and 26.3 ± 3.6 ng/mL (*n* = 10, Figure 2A). When pigs were euthanized in a random sequence on day 9, control pigs had low IGF-1 values (19.9 ± 2.6 ng/mL, *n* = 15, Figure 2B), while pigs treated with rhIGF-1 had plasma IGF-1 levels within the desired physiological range at 1–6.5 h after the last injection (91.0 ± 11.8 ng/mL, *n* = 20, Figure 2B).

#### Clinical assessments

No differences were found between rhIGF-1 and control pigs in baseline conditions such as birth weight (945 ± 41 vs. 938 ± 41 g), rectal temperature at 1 h (37.0 ± 0.2 vs. 37.0 ± 0.2) and 24 h after birth (38.1 ± 0.2 vs. 38.5 ± 0.2). The treatment had no effect on clinical score. Fecal score increased throughout the study period with no consistent differences between groups (Supplemental Table 6).

Twelve rhIGF-1 pigs and 19 control pigs were euthanized or died ahead of the pre-determined end of the experiment at day 9 (*p* = 0.27). The symptoms leading to early euthanasia were respiratory failure, severe NEC symptoms or poor tissue perfusion of hind legs reflected by development of bluish/grayish tissue color (four control pigs). One rhIGF-1 pig was euthanized after blood loss due to a disconnected PN line. The remaining pigs (21 rhIGF-1 pigs and 15 control pigs) were euthanized as planned on day 9. At tissue collection, the small intestinal weight of rhIGF-1 treated pigs was 20% higher than in control pigs, with both the proximal and distal regions affected (all *p* < 0.05). The rhIGF-1 pigs also showed a tendency to increased small intestine length (+11%, *p* = 0.09) and spleen weight (+26%, *p* = 0.08, Table 1). Blood biochemistry showed an increase in gamma-glutamyltransferase (GGT) and sodium levels in the blood of rhIGF-1 pigs compared with control pigs (both *p* < 0.05, Table 2).

**Table 1:** Body and organ weights of preterm pigs treated with rhIGF-1 or vehicle.

**Parameter**	**Exp. 1, rhIGF-1**	**Exp.1, Controls**	***p-*value**	**Exp. 2, rhIGF-1**	**Exp. 2, Controls**	***p-* value**
Number of animals^*^	23–25	24–25		17–18	16–18	
Kill weight, g	1,264 ± 78	1,208 ± 58	ns	994 ± 65	1025 ± 60	ns
Daily gain, g	43.5 ± 3.0	40.5 ± 4.4	ns	28 ± 3	31 ± 2	ns
SI total, g/kg	30.0 ± 1.3	25.1 ± 1.0	0.002	29.8 ± 0.9	28.6 ± 0.9	ns
SI prox, g/kg	9.4 ± 0.5	8.1 ± 0.4	0.04	9.7 ± 0.3	9.7 ± 0.4	ns
SI mid, g/kg	8.1 ± 0.7	7.0 ± 0.4	ns	10.1 ± 0.8	10.0 ± 0.7	ns
SI dist, g/kg	10.4 ± 0.5	8.2 ± 0.3	<0.001	9.5 ± 0.4	9.1 ± 0.3	ns
SI length, cm/kg	295 ± 13	266 ± 11	0.09	316 ± 15	309 ± 14	ns
Liver, g/kg	39.4 ± 1.1	41.6 ± 1.0	ns	28.2 ± 0.9	30.4 ± 1.3	ns
Spleen, g/kg	3.4 ± 0.3	2.7 ± 0.2	0.08	2.3 ± 0.1	1.8 ± 0.1	0.001
Kidney, g/kg	10.5 ± 0.4	10.3 ± 0.4	ns	10.3 ± 0.3	9.3 ± 0.4	ns
Stomach, g/kg	7.1 ± 0.2	7.2 ± 0.2	ns	8.5 ± 0.4	9.0 ± 0.6	ns

* Only pigs surviving until day 5 or beyond in Experiment 1 (longer term subcutaneous study) and until day 4 or beyond in Experiment 2 (short term continuous intra-arterial study) were included in the analyses. Values are mean ± SEM. Exp, experiment; ns, non-significant; SI, small intestine; prox, proximal; mid, middle; dist, distal.

**Table 2:** Plasma biochemistry of preterm pigs treated with rhIGF-1 or vehicle formulation.

**Parameter**	**Exp. 1, rhIGF-1**	**Exp. 1, Controls**	***p-* value**	**Exp. 2, rhIGF-1**	**Exp. 2, Controls**	***p-* value**
Number of animals	19	14		18	16–17	
Albumin g/l	17.5 ± 0.8	17.6 ± 0.9	ns	10.8 ± 0.4	10.7 ± 0.2	ns
Total protein g/l	36.0 ± 1.4	35.2 ± 1.7	ns	23.7 ± 2.0	23.0 ± 2.2	ns
Alkaline/basic phosphatase, U/l	1,465 ± 201	1,052.2 ± 178.9	ns	1,436 ± 220	1,391 ± 200	ns
Alanine aminotransferase, U/l	18.5 ± 1.0	16.7 ± 1.0	ns	15.1 ± 1.4	16.7 ± 1.2	ns
Aspartate aminotransferase, U/l	32.2 ± 2.8	32.4 ± 2.8	ns	33.7 ±10.9	37.6 ± 12.2	0.05
Bilirubin, μmol/l	3.6 ± 0.7	2.8 ± 0.4	ns	3.3 ± 0.8	2.6 ± 0.3	ns
Cholesterol, mmol/l	1.9 ± 0.2	1.8 ± 0.2	ns	1.8 ± 0.1	1.9 ± 0.1	ns
Creatine kinase, U/l	263 ± 40	226 ± 26	ns	147 ± 20	192 ± 19	0.01
Creatinine, μmol/l	35.3 ± 1.8	39.5 ± 4.8	ns	38.4 ± 1.4	43.7 ± 1.5	0.02
Iron, μmol/l	7.6 ± 1.2	9.0 ± 1.4	ns	9.3 ± 0.9	8.4 ± 0.9	ns
Inositol phosphate, mmol/l	1.1 ± 0.1	1.1 ± 0.1	ns	0.9 ± 0.0	0.9 ± 0.1	ns
Blood urea nitrogen, mmol/l	1.8 ± 0.3	1.5 ± 0.3	ns	0.96 ± 0.1	0.92 ± 0.1	ns
Gamma-glutamyltransferase, U/l	44.8 ± 4.4	32.6 ± 4.1	0.03	29.6 ± 3.0	33.0 ± 2.6	ns
Sodium, mmol/l	137.2± 0.8	134.9 ± 0.8	0.006	142.7± 0.6	142.9 ± 0.6	ns
Potassium, mmol/l	4.5 ± 0.2	4.5 ± 0.2	ns	4.7 ± 0.1	5.0 ± 0.1	0.03
Chlorid, mmol/l	95.9 ± 0.8	94.7 ± 1.2	ns	103.2± 0.5	103.9 ± 0.8	ns
Glucose hexokinase, mmol/l	11.4 ± 1.9	10.6 ± 1.9	ns	7.5 ± 0.7	7.9 ± 1.4	ns

Blood samples were collected on day 9 in Experiment 1 (longer term subcutaneous study) and on day 5 in Experiment 2 (short term continuous intra-arterial study). Values are mean ± SEM; Exp, experiment; ns, non-significant.

#### Hematology and immunological parameters

No differences in hematological values were observed between rhIGF-1 pigs and control pigs on day 9 (Table 3). TNF-α increases in response to LPS stimulation were highly variable and showed no difference between rhIGF-1 pigs (28.7 ± 16.9%, *n* = 15) and control pigs (60.2 ± 47.5%, *n* = 14). FACS analysis showed that the phagocytic activity and phagocytic capacity of neutrophils were similar between rhIGF-1 pigs (93.8 ± 1.1% and 3.8 ± 0.0 log (MFI), respectively, *n* = 19) and control pigs (94.2 ± 1.3% and 3.8 ± 0.1 log (MFI), respectively, *n* = 15). Also, no differences were observed between rhIGF-1 pigs (*n* = 19) and control pigs (*n* = 15) in %T cells of lymphocytes (35.4 ± 2.7 vs. 36.0 ± 4.0), %Th cells of T cells (54.5 ± 1.3 vs. 52.7 ± 2.0), %Tc cells of T cells (4.1 ± 0.4 vs. 4.4 ± 0.5) or %Treg cells of T cells (5.8 ± 0.8 vs. 5.5 ± 1.1). Consistently, no differences were observed in the total number (billions/L) of T cells (1.2 ± 0.3 vs. 1.4±0.2), Th cells (1.8 ± 0.3 vs. 2.0 ± 0.3), Tc cells (0.1 ± 0.0 vs. 0.2 ± 0.0), or Treg (0.2 ± 0.0 vs. 0.2 ± 0.0) in blood from rhIGF-1 pigs (*n* = 19) and control pigs (*n* = 13).

**Table 3:** Hematology of preterm pigs treated with rhIGF-1 or vehicle formulation subcutaneously for 8 days in Experiment 1.

**Parameter**	**rhIGF-1**	**Controls**	***p-*value**
Number of animals	19	13	
Total leukocytes, 10^9^/L	11.1 ± 1.3	12.5 ± 1.8	ns
Erythrocytes, 10^9^/L	3.0 ± 0.2	2.8 ± 0.2	ns
Thrombocytes, 10^9^/L	161.7 ± 18.6	218.2 ± 31.7	ns
Neutrophils, 10^9^ /L	6.4 ± 1.0	7.3 ± 1.4	ns
Lymphocytes, 10^9^/L	3.3 ± 0.5	3.7 ± 0.4	ns
Monocytes, 10^9^/L	0.17 ± 0.03	0.24 ± 0.07	ns
Eosinophils, 10^9^/L	1.0 ± 0.4	1.0 ±0.4	ns
Basophiles, 10^9^/L	0.05 ± 0.02	0.04 ± 0.02	ns
LUC, 10^9^/L	0.10 ± 0.01	0.14 ± 0.03	ns
Hemoglobin, mmol, L	3.7 ± 0.2	3.5 ± 0.2	ns
Hematocrit, L/L	0.19 ± 0.01	0.18 ± 0.01	ns
MCV, ft	65.0 ± 0.9	64.2 ± 1.1	ns
MCHC, mmol/L	19.5 ± 0.2	19.5 ± 0.2	ns
Mean platelet volume, ft	15.0 ± 0.6	15.2 ± 0.5	ns
Mean platelet count, g/L	235.4 ± 2.0	231.1 ±1.5	ns
Neutrophils, %	55.3 ± 3.4	55.3 ± 4.3	ns
Lymphocytes, %	34.0 ± 3.9	34.4 ± 4.3	ns
Monocytes, %	1.7 ± 0.2	2.0 ± 0.5	ns
Eosinophils, %	7.6 ± 2.8	6.7 ± 1.7	ns
Basophils, %	0.5 ± 0.2	0.3 ± 0.1	ns
LUC, %	1.0 ± 0.1	1.3 ± 0.3	ns

Blood samples were collected on day 9. Values are mean ± S EM. MCV, mean corpuscular volume. MCHC, mean corpuscular hemoglobin concentration; LUC, large unstained cells; Exp, experiment; ns, non-significant.

#### Glucose tolerance test and basal insulin and glucose levels

**Figure 3:**
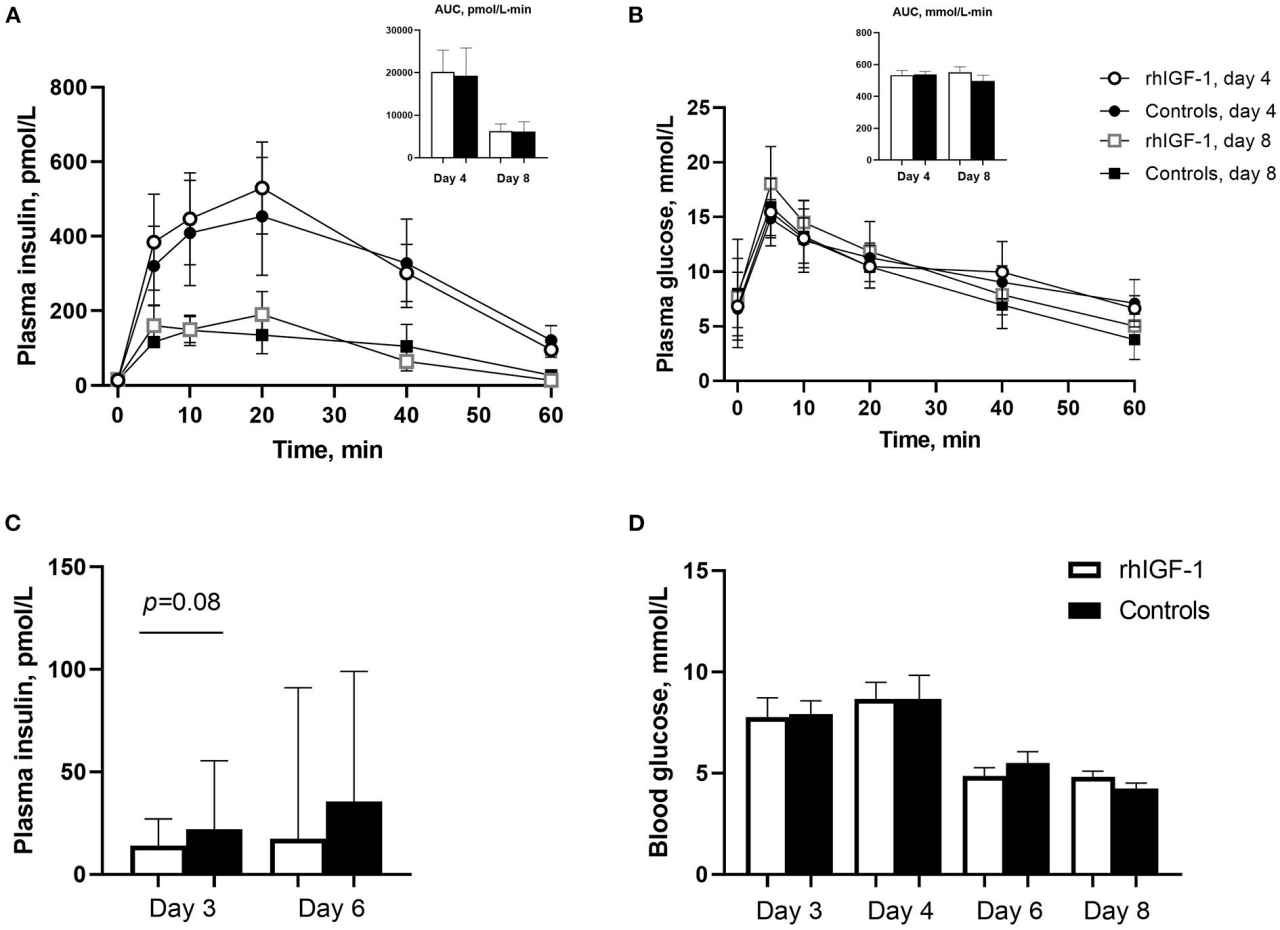
Blood insulin and glucose levels in Experiment 1. Plasma insulin **(A)** and glucose **(B)** levels during glucose tolerance test in rhIGF-1 and control preterm pigs day 4 (*n* = 11) and 8 (*n* = 6–7). **(C)** Basal plasma levels of insulin in rhIGF-1 and control preterm pigs day 3 (*n* = 25) and 6 (*n* = 20–23). **(D)** Blood glucose levels in rhIGF-1 and control preterm pigs day 3 (*n* = 24–25), 4 (*n* = 24–25), 6 (*n* = 22–23) and 8 (*n* = 14–18). rhIGF-1, recombinant human insulin-like growth factor-1; AUC, area under the curve. Values in **(A,B,D)** are presented as mean ± SEM. Values in **(C)** are presented as median ± IQR.

Plasma insulin and glucose responses to IAGTT were similar among groups on day 4 and 8 (Figures 3A,B). Compared with day 4, the area under the curve of insulin levels was significantly reduced at day 8, indicating a degree of insulin resistance in the preterm pigs on day 4 (*p* = 0.01, Figure 3A). Basic insulin and blood glucose did not differ significantly between groups (Figures 3C,D), but a tendency toward reduced insulin levels was observed on day 3 in rhIGF-1 pigs (*p* = 0.08). Large individual variation in plasma insulin levels was observed, particularly on day 6, with levels ranging 13.9–502 pmol/L in the rhIGF-1 pigs and 13.9–327 pmol/L in the control pigs (Figure 3C). On days 3–4, the high-glucose PN induced a moderate hyperglycemia (mean values ~8 mmol/L) relative to normal glucose values in suckling term pigs at 4–5 mmol/L (41, 63). On day 6–8, the glucose levels decreased to normal levels (mean values ~5 mmol/L), potentially due to improved glucose tolerance in the pigs combined with the decreasing volume of PN glucose at this time of the experiment.

#### NEC lesions

**Figure 4:**
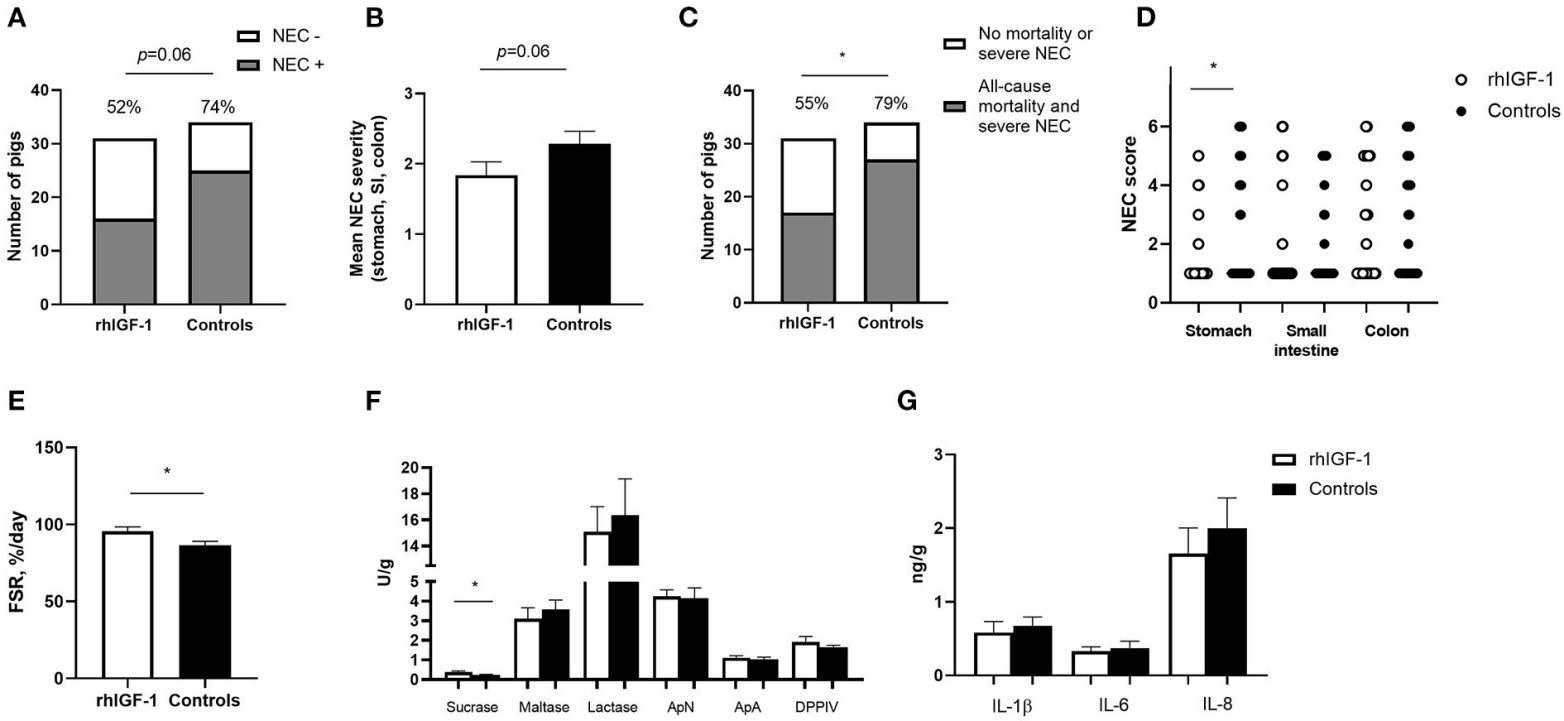
Intestinal parameters in Experiment 1. **(A)** Incidence of NEC (score ≥ 3 in at least one gut region) day 2–9 in rhIGF-1 and control preterm pigs (*n* = 31–34). **(B)** Average NEC severity score across gut regions in rhIGF-1 and control preterm pigs day 2–9 (*n* = 31–34). **(C)** Incidence of all-cause mortality and severe NEC day 2–9 (*n* = 31–34). **(D)** Individual NEC score distribution in stomach, small intestine (highest score of proximal, middle and distal section) and colon in rhIGF-1 and control preterm pigs day 2–9. **(E)** Fractional protein synthesis rate (FSR) in the distal small intestine of rhIGF-1 (*n* = 19) and control (*n* = 14) preterm pigs. **(F)** Brush border enzyme activities in proximal small intestine (*n* = 23–24). **(G)** Pro-inflammatory cytokines in colon (*n* = 22–24). rhIGF-1, recombinant human insulin-like growth factor-1; NEC, necrotizing enterocolitis; ApN, aminopeptidase N; ApA, aminopeptidase A; DPPIV, dipeptidylpeptidase; IL, interleukin. Values are mean ± SEM. *p < 0.05.

Across the 2–9 day period, overall incidence of NEC (52%, 16/31 vs. 74%, 25/34, *p* = 0.06, Figure 4A) and the average NEC severity across the entire gut tended to be lower in rhIGF-1 pigs than in control pigs, but the differences did not reach statistical significance (1.9 ± 0.2 vs. 2.3 ± 0.2, *p* = 0.06, Figure 4B). The rhIGF-1 pigs showed a reduced combined incidence of all-cause mortality (day 2-8) and severe NEC on day 9 (55%, 17/31 vs. 79%, 27/34, *p* < 0.05, Figure 4C). Colon, stomach, and small intestine NEC lesions were present in 28 (68%), 16 (39%), and 14 (34%) out of the total 41 NEC cases in the study, respectively, thus all gut regions were involved in NEC development, with colon lesions occurring most frequently. NEC lesions in the stomach region were reduced in rhIGF-1 pigs versus control pigs (*p* < 0.05), whereas no significant differences were observed in NEC scores in small intestine and colon between groups (Figure 4D).

#### Gut protein synthesis, morphology, function and inflammation

Protein synthesis in the distal small intestine was higher in rhIGF-1 than control pigs (+11%, 95.8 ± 2.7 vs. 86.6 ± 2.5, *n* = 14–19, *p* < 0.05, Figure 4E). No differences were observed between rhIGF-1 pigs and control pigs for villus heights (388 ± 27 vs. 396 ± 31 μm), crypt depths (84 ± 2 vs. 83 ± 2 μm), villus:crypt ratio (4.6 ± 0.3 vs. 4.8 ± 0.4), caspase-3 density (0.85 ± 0.42 vs. 0.79 ± 0.32%) or ki-67 density (32.8 ± 4.7 vs. 30.0 ± 3.3%) in the proximal small intestine (*n* = 19–20). This conclusion did not change after removing pigs with NEC lesions specifically in the proximal small intestine.

rhIGF-1 treated pigs had significantly increased levels of sucrase activity in the proximal region of the small intestine, compared with control pigs (*p* < 0.05, Figure 4F), whereas no differences were observed between groups in brush border enzymes in the distal region of the small intestine (data not shown). The levels of pro-inflammatory cytokines in colon were similar between rhIGF-1 and control pigs (Figure 4G) and no differences were observed for intestinal permeability (urinary lactulose/mannitol ratio, rhIGF-1: 0.13 ± 0.06, *n* = 15 vs. controls: 0.08 ± 0.03, *n* = 14). Likewise, the groups did not differ in density of bacteria detected in the bone marrow (logarithm of number colony-forming units: 3.98 ± 0.37, *n* = 21 vs. 3.82 ± 0.40, *n* = 19, respectively).

### Experiment 2: Short-term continuous systemic rhIGF-1 administration

#### Clinical values and circulating IGF-1 levels

**Figure 5:**
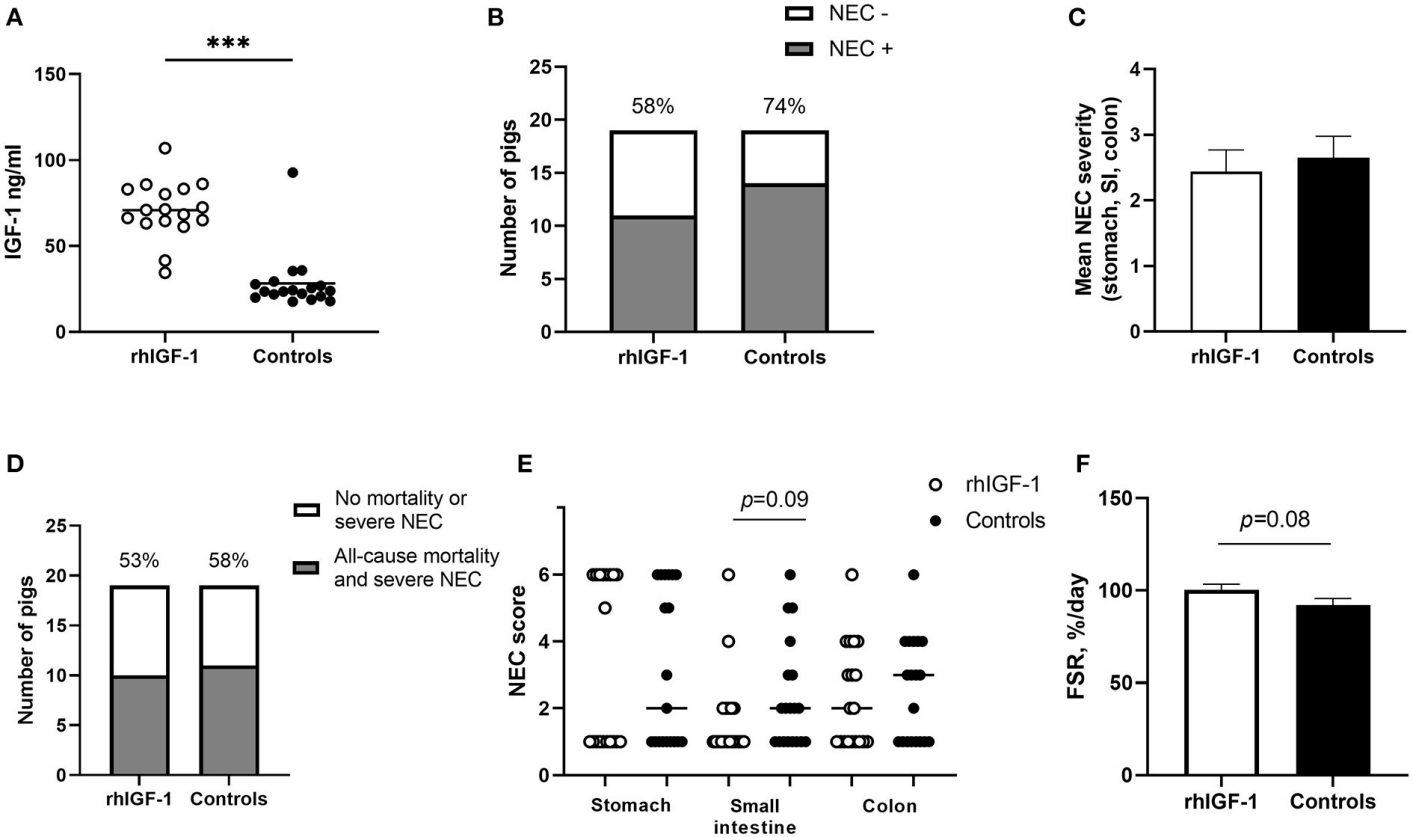
IGF-levels and intestinal parameters in Experiment 2. **(A)** Circulating IGF-1 levels at day 5 among rhIGF-1 pigs and control pigs (*n* = 17–18). **(B)** Incidence of NEC (score ≥ 3 in at least one gut region) day 2–5 in rhIGF-1 and control preterm pigs (*n* = 19). **(C)** Average NEC severity score across gut regions in rhIGF-1 pigs and control pigs day 2–5 (*n* = 19). **(D)** Incidence of all-cause mortality and severe NEC, day 2–5 (*n* = 19). **(E)** Individual NEC score distribution in stomach, small intestine (highest score of proximal, middle and distal section) and colon in rhIGF-1 and control preterm pigs day 2–5. F) Fractional protein synthesis rate (FSR) in the distal small intestine of rhIGF-1 (*n* = 17) and control (*n* = 14) preterm pigs. rhIGF-1, recombinant human insulin-like growth factor-1; NEC, necrotizing enterocolitis; SI, small intestine. Values in **(A)**, **(D)** and **(F)** are means ± SEM. ***p < 0.001.

No differences were found between rhIGF-1 pigs and control pigs in baseline conditions such as birth weight (876 ± 52 vs. 890 ± 54 g) or rectal temperature at 1 h (36.8 ± 0.1 vs. 36.5 ± 0.2) and 24 h after birth (37.5 ± 0.1 vs. 37.7 ± 0.1) (Supplemental Table 7). No consistent differences in clinical and fecal score were noted between groups and no differences in overall mortality were found. One control pig was euthanized on day 2 because of clinical NEC symptoms and one rhIGF-1 pig was euthanized on day 3 because of catheter-related problems. The remaining pigs (18 pigs/group) were euthanized as planned on day 5 with body and organ weights shown in Table 1. Weight gain and organ weights were similar between groups, except for the spleen, which showed increased weight in rhIGF-1 pigs (+28%, *p* = 0.001). Further, plasma levels of creatine kinase, creatinine, aspartate aminotransferase and potassium were reduced in rhIGF-1 pigs (all *p* ≤ 0.05, Table 2). There was no difference in blood glucose on day 3 (3.5 ± 0.3 vs. 4.2 ± 0.3 mmol/L). Basal insulin levels could not be detected by our assay, neither on day 3 or 5, in neither of the groups (data not shown). At euthanasia day 5, plasma IGF-1 levels were higher in rhIGF-1 than control pigs (70.9 ± 4.1, *n* = 17 vs. 27.7 ± 4.2 ng/mL, *n* = 18, Figure 5A).

#### Hematology and immunological parameters

No differences in hematological values were observed between rhIGF-1 pigs and control pigs on day 5 (Table 4). Also, FACS analysis showed no significant difference in phagocytic activity and phagocytic capacity of neutrophils (rhIGF-1: 71.5 ± 6% and 4.3 ± 0.0 log (MFI), *n* = 18 vs. controls: 65.8 ± 6% and 4.3 ± 0.0 log (MFI), *n* = 17). For TLR4 stimulation of whole blood, no difference in TNF-α levels was observed between the groups.

**Table 4:** Hematology of preterm pigs treated with rhIGF-1 or vehicle formulation, continuous intra-arterially for 4 days in Experiment 2.

**Parameter**	**rhIGF-1**	**Controls**	***p-*value**
Number of animals	18	16	
Total leukocytes, 10^9^/L	2.2 ± 0.1	2.3 ± 0.2	ns
Immature neutrophils, 10^9^/L	0.20 ± 0.1	0.15 ± 0.1	ns
Neutrophils, 10^9^/L	0.93 ± 0.1	1.10 ± 0.2	ns
Lymphocytes, 10^9^/L	1.01 ± 0.1	0.97 ± 0.1	ns
Monocytes, 10^9^/L	0.05 ± 0.0	0.06 ± 0.0	ns
Basophiles, 10^9^/L	0.00 ± 0.0	0.01 ± 0.0	ns
Eosinophils, 10^9^/L	0.02 ± 0.0	0.02 ± 0.0	ns
Erythrocytes, 10^9^/L	3.4 ± 0.1	3.5 ± 0.1	ns
Thrombocytes, 10^9^/L	115.4 ± 11.6	115.8 ± 12.3	ns
Hemoglobin, mmol, L	4.5 ± 0.1	4.6 ± 0.1	ns
Hematocrit, L/L	0.24 ± 0.0	0.25 ± 0.0	ns
MCV, ft	71.0 ± 0.6	71.2 ± 0.9	ns
MCHC, mmol/L	18.3 ± 0.1	18.5 ± 0.2	ns
Reticulocytes, %	4.6 ± 0.4	4.1 ± 0.3	ns
Reticulocytes, 10^9^/L	159.6 ± 13.6	142.9 ± 9.3	ns

Blood samples were collected on day 5. Values are mean ± SEM. MCV, mean corpuscular volume; MCHC, mean corpuscular hemoglobin concentration; ns, non-significant.

#### Gut NEC lesions, morphology, function and inflammation markers

Across the 2–5 day period, NEC incidence (rhIGF-1: 58%, 11/19 vs. controls: 74%, 14/19, Figure 5B) and the average NEC score across the gut (2.4 ± 0.3 vs. 2.7 ± 0.3, Figure 5C) did not differ between groups. Likewise, the combined incidence of all-cause mortality (day 2–4) and severe NEC on day 5 (53%, 10/19 vs. 58%, 11/19, Figure 5D) did not differ between groups. Colon, stomach, and small intestine NEC lesions were present in 18 (72%), 18 (72%) and 8 (32%) of the total 25 NEC cases. rhIGF-1 treated pigs tended to have reduced NEC scores in the small intestine (*p* = 0.09, Figure 5E).

The mean fractional protein synthesis rate in the distal small intestine was 9% higher in rhIGF-1 pigs compared with controls (100.4 ± 3.0 vs. 92.1 ± 3.6, *n* = 14–17/group, *p* = 0.08, Figure 5F). There were no differences in villus height (525 ± 30 vs. 524 ± 35 μm), crypt depth (118 ± 6 vs.111 ± 5 μm) or villus to crypt ratio (4.7 ± 0.4 vs. 4.5 ± 0.3) in the proximal small intestine of rhIGF-1 and control pigs, respectively. Removing pigs with NEC in the proximal intestine did not change this conclusion. No differences were observed in lactulose/mannitol ratio (0.12 ± 0.03 vs. 0.09 ± 0.02, *n* = 11–17) and activities of gut enzymes (sucrase, maltase, lactase, ApN, ApA, and DPPIV) in proximal and distal small intestine. Likewise, no differences in IL-1β, IL-6 and IL-8 levels in the colon were observed.

## Discussion

The association between circulating IGF-1 levels and a range of morbidities in preterm infants related to gut, lungs, bones and brain is well-documented (5–8, 12, 14, 15, 42). Yet, there is limited evidence of direct effects on such morbidities by supplementing IGF-1 during the first weeks after preterm birth. Thus, it is important to investigate the preclinical safety and efficacy of IGF-1 supplementation using clinically relevant administration methods and appropriate animal models of preterm infants. Preterm pigs delivered at 90% gestation show morbidities similar to those in many preterm infants, including reduced growth (41), impaired postnatal gut, brain and immune development (32–34, 41, 43, 44, 60, 64), neonatal hypoglycemia (41, 63) and reduced IGF-1 levels (41, 42), relative to their term-born counterparts. In this study, preterm pigs treated with rhIGF-1 s.c. for 8 days achieved circulating IGF-1 levels similar to those in term suckling pigs (42), reduced incidence of severe complications (reflected by the combined incidence of all-cause mortality day 2-8 and severe NEC on day 9), and increased intestinal weight and protein anabolic response. There were limited effects on a large series of measured gut structural, functional and immunological indices as well as on blood chemistry, hematology, glucose homeostasis and systemic immunity development. Collectively, the findings support our previous 5-day study (42) and other *in vivo* animal studies demonstrating increased intestinal mucosal growth after parenteral (65) and especially oral IGF-1 administration (35, 36, 66, 67), as well as the reduced NEC incidence in a rodent study (68).

In the present study, only the longer-term rhIGF-1 s.c. supplementation for 8 days improved gut growth in preterm neonates and tended to reduce NEC. Continuous systemic administration of rhIGF-1 for just 4 days had limited effects on NEC and gut parameters, contrasting the more clear effects in our earlier study, using twice daily s.c. administrations (42). Yet, the plasma IGF-1 levels appeared to be within the desired physiological range in both studies. Treatment effects may depend on the mode, dose and length of IGF-1 administration, and studies are required to know if different methods of IGF-1 supplementation during the first week(s) after preterm birth exert differential short- and long-term effects on the maturation of the gut as well as other organs. The reason why our continuous, intra-arterial infusion failed to demonstrate efficacy on stimulating gut parameters is currently unknown. Possibly, intermittent rhIGF-1 dosing is most efficacious for physiological responses at receptor levels in the gut of immature newborns. Also, we cannot exclude the possibility that the active rhIGF-1 drug interacted with components of the PN solution, negatively affecting its physiological effects.

Considering the trophic effect on the intestine after 8 days of s.c. rhIGF-1 treatment, it was surprising that IGF-1 did not affect mucosal structure, function or permeability. Also, we were unable to demonstrate proliferative or anti-apoptotic effects on the small intestinal epithelium, as tested by immunohistochemical staining. Possibly, longer-term, supra-physiological, IGF-1 administration is required to induce more robust effects on mucosal growth and function, acting *via* IGF-1 receptors on the intestinal epithelial cells (69). Evidence suggests that there may be only early and transient changes in intestinal crypt and villi morphology in response to trophic pharmacological therapies and that IGF-1 preferentially may target intestinal epithelial stem cells (IESC), located at the crypt base (70). Thus, IESC expansion and subsequent crypt fission (i.e., synthesis of new crypts) may reflect the adaptive growth and response to IGF-1 therapy more accurately (70). Beyond the gut, IGF-1 treatment did not appear to change the clinical status of the pigs, except for few indications of mild effects on biochemical markers of muscle, liver and kidney function. None of the findings were consistent across the current two experiments or our previous intervention study (42) and the levels were in the normal reference range of preterm pigs (e.g., aspartate aminotransferase, creatinine, sodium, potassium) (71, 72) or weaning farm piglets (e.g., gamma-glutamyltransferase) (73). Because local and systemic IGF-1 exposure potentially plays a role for liver, kidney, lung, bone and brain development and function (5–8, 12, 14, 15, 74, 75), we have recently conducted a longer-term study in preterm pigs to investigate if supplemental rhIGF-1 for 18 days affects these organs. By postnatal day 19, overall mortality was again reduced and spleen weight increased in rhIGF-1 treated pigs, whereas the biochemical profile was similar between groups (unpublished observations). Detailed organ-related and immune analyses from this study will be reported as a separate publication. In both infants and pigs, treatment effects may be highly organ-specific and local IGF-1 levels and receptor-mediated actions in the tissues may not necessarily reflect circulating IGF-1 levels. In this regard, preterm pigs are a valuable tool to test drug delivery procedures (enteral, parenteral, bolus, continuous, s.c., intravenous, intraperitoneal) for preterm infants and to investigate the pharmacodynamics of rhIGF-1, together with cell- and tissue-specific effects.

Disturbed glucose homeostasis is a common complication in preterm infants (54) and a clinical trial indicated improved glucose metabolism and tolerance after rhIGF-1 treatment (18). On the other hand, it is critical that rhIGF-1 treatment does not lead to hypoglycemia. In our study, basal glucose levels and plasma glucose and insulin responses to IAGTT, were similar between rhIGF-1 and control pigs. Some insulin resistance was evident in preterm pigs during the first 4–5 days of life as indicated by the higher insulin secretory response after IAGTT. This finding is consistent with other recent reports showing that preterm pigs are insulin resistant, compared with term pigs (76, 77). Later, insulin sensitivity and glucose tolerance were improved, probably due to maturation of peripheral insulin signaling, glucose transport and pancreatic β-cell function. Previous studies showed improved insulin sensitivity in neonatal pigs fed intermittent enteral formula compared with continuous PN (78, 79). Thus, improved insulin sensitivity in preterm pigs may also be associated with a reduction in continuous PN supply and increase in intermittent EN feeding throughout the study. In this context, the high mortality observed in Experiment 1 may partly result from hyperglycemia in response to the relatively high loads of glucose supplied *via* the PN. As previously described for critically ill adults and preterm infants, excessive supply of glucose *via* PN may lead to increased mortality and sepsis (55–57). The mechanisms behind such glucose-induced toxicity is not clear, but can probably be explained by glucose overload, mitochondrial dysfunction and oxidative phosphorylation in cellular systems with passive, insulin-independent glucose uptake, such as neurons, hepatocytes, endothelial and immune cells (80). Several studies have also shown an association between hyperglycemia and impaired innate immunity (80). We cannot exclude that the serious tissue perfusion problems observed in four control pigs (euthanized ahead of time) were also related to high glucose load but this, and the possible effects of rhIGF-1 supplementation to prevent glucose-related adverse symptoms, remain to be further investigated.

Like infants, pigs born preterm have impaired bacterial phagocytosis and pro-inflammatory responses related to innate immune defects (43, 44, 81, 82) and both species have high susceptibility to infection and bacteremia (17, 83–85). The role of IGF-1 in systemic immune development and function is complex and results are partly contradicting. The IGF-1 receptor (IGF-1R) is expressed on various leukocyte populations and IGF-1 supports differentiation, proliferation, survival and metabolism of T cell, B cell, granulocytic, and monocytic lineages and immunoglobulin production and class-switching (86–88). In neonates, IGF-1 promotes phytohemagglutinin (PHA)-induced proliferation, maturation, survival and IL-6 expression in cord blood mononuclear cells (CBMC) (86, 87). However, IGF-1 also displays anti-inflammatory properties suppressing IFN-γ expression and DNA binding activity of transcription factor nuclear factor-κB (NF-κB) and activator protein-1 (AP-1) in stimulated CBMC (89) and the TLR4/NF-κB pathway in ileum of a neonatal NEC rat model (68). This supports that rhIGF-1 treatment reduced mean blood TNF-α production after *in vitro* stimulation by ~50% in Experiment 1 (although the difference did not reach statistical significance) and also reduced basal blood IFN-γ and TNF-α expressions in our recent 19 day study (unpublished observations). A reduced immune response may potentially increase sepsis sensitivity. Effects of IGF-1 treatment on infant sepsis in the study of Ley et al. (18) were not significant (38 vs. 25% in controls), but possible immune effects of IGF-1 treatment warrant further study.

In preterm pigs, the leukocyte and neutrophil numbers, as well as phagocytic capacity, increase sharply in the second week of life, as part of a natural physiological maturation of the immune system (43). Interestingly, rhIGF-1 treatment increased the spleen weight both on day 5 and day 9, but we were unable to demonstrate any systemic immune effects by hematology, FACS or *in vitro* analysis of the blood. Accordingly, Clark et al. (88) showed that adult mice treated with continuous s.c. IGF-1 for 7 and 14 days had markedly increased spleen, thymus and kidney weights. The increased spleen weight was shown to be due to a pronounced increase in T cells and B cell numbers. However, among peripheral blood leukocytes only neutrophil counts were significantly increased. Clearly, there is a need to better understand the effects of supplemental rhIGF-1 on the primary and secondary lymphoid organs in preterm neonates following different stages of immaturity at birth and postnatal ages.

The highly sensitive preterm pig model enables preclinical studies of many clinically relevant topics and interventions that cannot be examined in preterm infants because of low incidence of selected diseases or ethical reasons. On the other hand, direct translation between infants and piglets, and across organs, is difficult due to species- and organ-specific maturational patterns (90). Thus, the nature and timing of spontaneous organ lesions, including NEC, may differ between 90% gestation preterm pigs and 60–80% gestation preterm infants. Yet, the high sensitivity to sepsis and NEC lesions in formula-fed preterm pigs, immature blood and tissue immune systems, and moderately immature brain structure/functions, make preterm pigs a useful model for very preterm infants within many areas of neonatology, with opportunities to control several external factors and access to all organs using clinically-relevant procedures.

In conclusion, neither longer-term intermittent nor short-term continuous systemic IGF-1 supplementation showed any marked effects on clinical or paraclinical outcomes related to growth, metabolism and immunity. However, the findings did suggest a positive effect of IGF-1 supplementation on potential overall viability and gut maturation and growth, particularly after a longer period of s.c treatment. The effects on spleen weight and immune responses deserve further investigation. It is important to investigate longer-term effects of various forms of IGF-1 supplementation across a range of organs and the preterm pig may be an excellent model for this. Collectively, the results add further preclinical evidence for the safety and efficacy of supplemental IGF-1 to hospitalized extremely preterm infants.

## Data availability statement

The original contributions presented in the study are included in the article/Supplementary material, further inquiries can be directed to the corresponding author.

## Ethics statement

The animal study was reviewed and approved by the Danish Animal Experiments Inspectorate, Ministry of Environment and Food of Denmark.

## Author contributions

KH conceived and designed the experiments, acquired, analyzed and interpreted data, and wrote the paper. MR analyzed and interpreted data and wrote the paper. PTS conceived and designed the experiments, analyzed and interpreted data, helped to draft the paper, and took final responsibility for its contents. DB analyzed and interpreted data. TT and GC conceived and designed the experiments. All authors revised the manuscript critically for important intellectual content and approved the final version. All authors contributed to the article and approved the submitted version.

## Funding

The authors declare that this study received funding from Takeda, MA, USA. The funder was not involved in the study design, collection, analysis, interpretation of data, the writing of this article or the decision to submit it for publication.

## Conflict of interest

Author GC was employed by the company Takeda, MA, USA. Author PTS is currently involved in a patent application directed to use of rhIGF-1 for preterm infants. The remaining authors declare that the research was conducted in the absence of any commercial or financial relationships that could be construed as a potential conflict of interest.

## Publisher's note

All claims expressed in this article are solely those of the authors and do not necessarily represent those of their affiliated organizations, or those of the publisher, the editors and the reviewers. Any product that may be evaluated in this article, or claim that may be made by its manufacturer, is not guaranteed or endorsed by the publisher.
