# Supplementary material for: Clinical outcome and gut development after insulin-like growth factor-1 supplementation to preterm pigs
Source: Front Pediatr. 2022 Aug 5;10:868911. doi: 10.3389/fped.2022.868911 (PMC9389362; doi:10.3389/fped.2022.868911)
Supplement: Supplementary file 1 [file Data_Sheet_1.docx]

**Supplemental Table S1.** Parenteral and enteral composition

| Product | Amount (g/l) | Supplier |
| --- | --- | --- |
| Lacprodan DI-9224, whey protein | 55 | Arla Foods Ingredients |
| Calogen LCT, lipid emulsion | 30 | Nutricia, Allerød, Denmark |
| Liquigen MCT, lipid emulsion | 43 | Nutricia |
| Fantomalt | 25 | Nutricia |
| Phlexy-Vits, vitamin and mineral mix | 2 | Nutricia |
| Miprodan 40 | 25 | Arla Foods Ingredients |

Enteral composition

| Nutrient content | EN, Exp. 1 & 2 | PN, Exp. 1, day 1-2 | PN, Exp. 1, day 3-9 | PN, Exp. 2 |
| --- | --- | --- | --- | --- |
| Energy, kJ/L | 2947 | 3248-3564 | 3778-4028 | 3136 |
| Total AA, g/L | 70 | 40-42 | 35-37 | 42 |
| Carbohydrate, g/L | 24 | 160-180 | 140-159 | 91 |
| Fat, g/L | 37 | 0 | 24-26 | 29 |

Enteral and parenteral diet composition in Experiment 1 and 2. In Experiment 1, the lipid fraction of the parenteral nutrition was not added until day 3 and the amount of 50% glucose added to the PN bags was 300-440 ml. EN, Enteral nutrition; PN, parenteral nutrition; AA, amino acids; Exp, Experiment.

**Supplemental Table S2.** Total nutrient supply in Experiment 1

| Day | 1 | 2 | 3 | 4 | 5 | 6 | 7 | 8 | 9 |
| --- | --- | --- | --- | --- | --- | --- | --- | --- | --- |
| PN, mL/kg/d | 120 | 144 | 144 | 120-144 | 120-144 | 40-120 | 72-96 | 72-96 | 72-120 |
| EN, mL/kg/d | 32 | 32 | 48 | 64 | 64 | 80 | 80-96 | 80-96 | 80-96 |

Volumes of parenteral and enteral nutrition provided on different days throughout Experiment 1. PN, parenteral nutrition; EN, Enteral nutrition.

| Day | 1 | 2 | 3 | 4 | 5 | 6 | 7 | 8 | 9 |
| --- | --- | --- | --- | --- | --- | --- | --- | --- | --- |
| PN, energy, kJ/kg/d | 390-428 | 468-513 | 544-580 | 483-580 | 483-580 | 193-483 | 290-388 | 290-388 | 290-453 |
| EN, energy, kJ/kg/d | 94 | 94 | 142 | 187 | 187 | 236 | 236-283 | 236-283 | 236-283 |
| Total energy, kJ/kg/d | 484-522 | 562-607 | 686-722 | 670-767 | 670-767 | 429-719 | 573-671 | 573-671 | 573-689 |
| PN, AA, g/kg/d | 4.8-5.1 | 5.7-6.1 | 5.0-5.3 | 4.2-5.3 | 4.2-5.3 | 1.7-4.4 | 2.5-3.5 | 2.5-3.5 | 2.5-4.4 |
| EN, AA, g/kg/d | 2.2 | 2.2 | 3.4 | 4.5 | 4.5 | 5.6 | 5.6-6.7 | 5.6-6.7 | 5.6-6.7 |
| Total AA, g/kg/d | 7.0-7.3 | 7.9-8.3 | 8.4-8.7 | 8.7-9.8 | 8.7-9.8 | 7.3-10.0 | 9.2-10.2 | 9.2-10.2 | 9.2-10.0 |
| PN, glucose, g/kg/d | 19.2-21.7 | 23.0-26.0 | 20.1-22.8 | 19.0-22.8 | 19.0-22.8 | 7.6-19.0 | 11.4-15.2 | 11.4-15.2 | 11.4-16.7 |
| EN, carbohydrate, g/kg/d | 0.8 | 0.8 | 1.2 | 1.5 | 1.5 | 1.9 | 1.9-2.3 | 1.9-2.3 | 1.9-2.3 |
| Total carbohydrate, g/kg/d | 20.0-22.5 | 23.8-26.8 | 21.3-24.0 | 20.5-24.3 | 20.5-24.3 | 9.5-20.9 | 13.7-17.5 | 13.7-17.5 | 13.7-18.6 |
| PN, fat, g/kg/d | 0 | 0 | 3.5-3.7 | 2.9-3.7 | 2.9-3.7 | 1.2-3.1 | 1.7-2.4 | 1.7-2.4 | 1.7-3.1 |
| EN, fat, g/kg/d | 1.2 | 1.2 | 1.8 | 2.4 | 2.4 | 3.0 | 3.0-3.6 | 3.0-3.6 | 3.0-3.6 |
| Total fat, g/kg | 1.2 | 1.2 | 5.3-5.5 | 5.-6.1 | 5.3-6.1 | 4.2-6.1 | 5.3-6.0 | 5.3-6.0 | 5.3-6.1 |

Total nutrient supply by parenteral and enteral nutrition per kilogram of body weight per day in Experiment 1. The lipid fraction of the parenteral nutrition was not added until day 3 and the amount of 50% glucose added to the PN bags was 300-440 mL. EN, Enteral nutrition; PN, parenteral nutrition; AA, amino acids.

**Supplemental Table S3.** Total nutrient supply in Experiment 2.

| Day | 1 | 2 | 3 | 4 | 5 |
| --- | --- | --- | --- | --- | --- |
| PN, mL/kg/d | 120 | 120 | 120 | 120 | 120 |
| EN, mL/kg/d | 16 | 32 | 48 | 80 | 80 |

Volumes of parenteral and enteral nutrition provided on different days throughout Experiment 2. PN, parenteral nutrition; EN, Enteral nutrition.

| Day | 1 | 2 | 3 | 4 | 5 |
| --- | --- | --- | --- | --- | --- |
| PN, energy, kJ/kg/d | 376 | 376 | 376 | 376 | 376 |
| EN, energy, kJ/kg/d | 47 | 94 | 141 | 236 | 236 |
| Total energy, kJ/kg/d | 423 | 470 | 517 | 612 | 612 |
| PN, AA, g/kg/d | 5.0 | 5.0 | 5.0 | 5.0 | 5.0 |
| EN, AA, g/kg/d | 1.1 | 2.2 | 3.4 | 5.6 | 5.6 |
| Total AA, g/kg/d | 6.1 | 7.2 | 8.4 | 10.6 | 10.6 |
| PN, glucose, g/kg/d | 10.9 | 10.9 | 10.9 | 10.9 | 10.9 |
| EN, carbohydrate, g/kg/d | 0.4 | 0.8 | 1.2 | 1.9 | 1.9 |
| Total carbohydrate, g/kg/d | 11.3 | 11.7 | 12.1 | 12.8 | 12.8 |
| PN, fat, g/kg/d | 3.5 | 3.5 | 3.5 | 3.5 | 3.5 |
| EN, fat, g/kg/d | 0.6 | 1.2 | 1.8 | 3.0 | 3.0 |
| Total fat, g/kg/d | 4.1 | 4.7 | 5.3 | 6.5 | 6.5 |

Total nutrient supply by parenteral and enteral nutrition per kilogram of body weight per day in Experiment 2. EN, Enteral nutrition; PN, parenteral nutrition; AA, amino acids.

**Supplemental Table S4.** Plasma IGF-1 levels in each pig day 3, 5 and at euthanasia in Experiment 2.

| Pig ID | Litter | Treatment | Day | IGF-1 levels |  |
| --- | --- | --- | --- | --- | --- |
| 1900-19 | 1 | CON | 3 | 18 |  |
| 1900-19 | 1 | CON | 5 | N/A |  |
| 1900-19 | 1 | CON | Euth. | <10 |  |
| 1901-19 | 1 | CON | 3 | 26.5 |  |
| 1901-19 | 1 | CON | 5 | 32.5 |  |
| 1901-19 | 1 | CON | Euth. | 29.4 |  |
| 1902-19 | 1 | CON | 3 | 26,2 |  |
| 1902-19 | 1 | CON | 5 | 20,6 |  |
| 1902-19 | 1 | CON | Euth. | 20.8 |  |
| 1905-19 | 1 | CON | 3 | 18.4 |  |
| 1905-19 | 1 | CON | 5 | 18.2 |  |
| 1905-19 | 1 | CON | Euth. | 17.9 |  |
| 1906-19 | 1 | CON | 3 | 17,3 |  |
| 1906-19 | 1 | CON | 5 | 21.8 |  |
| 1906-19 | 1 | CON | Euth. | 21.9 |  |
| 1909-19 | 1 | CON | 3 | 19,8 |  |
| 1909-19 | 1 | CON | 5 | 22.2 |  |
| 1909-19 | 1 | CON | Euth. | 17.6 |  |
| 1903-19 | 1 | rhIGF-1 | 3 | 282 |  |
| 1903-19 | 1 | rhIGF-1 | 5 | 248 |  |
| 1903-19 | 1 | rhIGF-1 | Euth. | 71.3 |  |
| 1904-19 | 1 | rhIGF-1 | 3 | 277 |  |
| 1904-19 | 1 | rhIGF-1 | 5 | 207 |  |
| 1904-19 | 1 | rhIGF-1 | Euth. | 61.2 |  |
| 1907-19 | 1 | rhIGF-1 | 3 | 278 |  |
| 1907-19 | 1 | rhIGF-1 | 5 | 296 |  |
| 1907-19 | 1 | rhIGF-1 | Euth. | 72.4 |  |
| 1908-19 | 1 | rhIGF-1 | 3 | 197 |  |
| 1908-19 | 1 | rhIGF-1 | 5 | 128 |  |
| 1908-19 | 1 | rhIGF-1 | Euth. | 66.3 |  |
| 1910-19 | 1 | rhIGF-1 | 3 | 289 |  |
| 1910-19 | 1 | rhIGF-1 | 5 | 134 |  |
| 1910-19 | 1 | rhIGF-1 | Euth. | 41.6 |  |
| 1911-19 | 1 | rhIGF-1 | 3 | 458 |  |
| 1911-19 | 1 | rhIGF-1 | 5 | 93.9 |  |
| 1911-19 | 1 | rhIGF-1 | Euth. | 68.3 |  |
| 1912-19 | 1 | rhIGF-1 | 3 | 448 |  |
| 1912-19 | 1 | rhIGF-1 | 5 | 164 |  |
| 1912-19 | 1 | rhIGF-1 | Euth. | 64.6 |  |
| 1913-19 | 1 | rhIGF-1 | 3 | 302 |  |
| 1913-19 | 1 | rhIGF-1 | 5 | 194 |  |
| 1913-19 | 1 | rhIGF-1 | Euth. | 83 |  |
| 1914-19 | 1 | CON | 3 | 25,2 |  |
| 1914-19 | 1 | CON | 5 | 24.2 |  |
| 1914-19 | 2 | CON | Euth. | 23.8 |  |
| 1915-19 | 2 | CON | 3 | 28.6 |  |
| 1915-19 | 2 | CON | 5 | 23.2 |  |
| 1915-19 | 2 | CON | Euth. | 27.7 |  |
| 1918-19 | 2 | CON | 3 | 21.3 |  |
| 1918-19 | 2 | CON | 5 | 22.7 |  |
| 1918-19 | 2 | CON | Euth. | 25.5 |  |
| 1919-19 | 2 | CON | 3 | 27.3 |  |
| 1919-19 | 2 | CON | 5 | N/A |  |
| 1919-19 | 2 | CON | Euth. | 92.8 |  |
| 1922-19 | 2 | CON | 3 | 24,4 |  |
| 1922-19 | 2 | CON | 5 | 28.3 |  |
| 1922-19 | 2 | CON | Euth. | 35.5 |  |
| 1923-19 | 2 | CON | 3 | 31.3 |  |
| 1923-19 | 2 | CON | 5 | 20.5 |  |
| 1923-19 | 2 | CON | Euth. | 23.5 |  |
| 1926-19 | 2 | CON | 3 | 25.5 |  |
| 1926-19 | 2 | CON | 5 | NA |  |
| 1926-19 | 2 | CON | Euth. | 23.5 |  |
| 1927-19 | 2 | CON | 3 | 21.9 |  |
| 1927-19 | 2 | CON | 5 | 35.3 |  |
| 1927-19 | 2 | CON | Euth. | 35.8 |  |
| 1930-19 | 2 | CON | 3 | 21.8 |  |
| 1930-19 | 2 | CON | 5 | 27.6 |  |
| 1930-19 | 2 | CON | Euth. | 22.2 |  |
| 1931-19 | 2 | CON | 3 | 17,2 |  |
| 1931-19 | 2 | CON | 5 | 15.5 |  |
| 1931-19 | 2 | CON | Euth. | 24.2 |  |
| 1933-19 | 2 | CON | 3 | 20.5 |  |
| 1933-19 | 2 | CON | 5 | 23.2 |  |
| 1933-19 | 2 | CON | Euth. | 18.8 |  |
| 1934-19 | 2 | CON | 3 | 17,7 |  |
| 1934-19 | 2 | CON | 5 | 23.6 |  |
| 1934-19 | 2 | CON | Euth. | 26.8 |  |
| 1916-19 | 2 | rhIGF-1 | 3 | 283 |  |
| 1916-19 | 2 | rhIGF-1 | 5 | 213 |  |
| 1916-19 | 2 | rhIGF-1 | Euth. | N/A |  |
| 1917-19 | 2 | rhIGF-1 | 3 | 168 |  |
| 1917-19 | 2 | rhIGF-1 | 5 | 231 |  |
| 1917-19 | 2 | rhIGF-1 | Euth. | 34.4 |  |
| 1920-19 | 2 | rhIGF-1 | 3 | 295 |  |
| 1920-19 | 2 | rhIGF-1 | 5 | 219 |  |
| 1920-19 | 2 | rhIGF-1 | Euth. | 70.9 |  |
| 1921-19 | 2 | rhIGF-1 | 3 | 201 |  |
| 1921-19 | 2 | rhIGF-1 | 5 | 276 |  |
| 1921-19 | 2 | rhIGF-1 | Euth. | 85.8 |  |
| 1924-19 | 2 | rhIGF-1 | 3 | 164 |  |
| 1924-19 | 2 | rhIGF-1 | 5 | 161 |  |
| 1924-19 | 2 | rhIGF-1 | Euth. | 80.2 |  |
| 1925-19 | 2 | rhIGF-1 | 3 | 281 |  |
| 1925-19 | 2 | rhIGF-1 | 5 | 187 |  |
| 1925-19 | 2 | rhIGF-1 | Euth. | 83.3 |  |
| 1928-19 | 2 | rhIGF-1 | 3 | 303 |  |
| 1928-19 | 2 | rhIGF-1 | 5 | 226 |  |
| 1928-19 | 2 | rhIGF-1 | Euth. | 107 |  |
| 1929-19 | 2 | rhIGF-1 | 3 | 282 |  |
| 1929-19 | 2 | rhIGF-1 | 5 | 233 |  |
| 1929-19 | 2 | rhIGF-1 | Euth. | 86.1 |  |
| 1932-19 | 2 | rhIGF-1 | 3 | 143 |  |
| 1932-19 | 2 | rhIGF-1 | 5 | 255 |  |
| 1932-19 | 2 | rhIGF-1 | Euth. | 83.2 |  |
| 1935-19 | 2 | rhIGF-1 | 3 | 222 |  |
| 1935-19 | 2 | rhIGF-1 | 5 | N/A |  |
| 1935-19 | 2 | rhIGF-1 | Euth. | 65.1 |  |
| A | 2 | rhIGF-1 | 3 | 61.4 |  |
| A | 2 | rhIGF-1 | 5 | N/A |  |
| A | 2 | rhIGF-1 | Euth. | N/A |  |

Blood samples day 3 and 5 were taken via the umbilical catheter, blood samples at euthanasia (Euth.) day 5. rhIGF-1, recombinant human insulin-like growth factor-1; NA, not available.

**Supplemental Table S5.** Plasma IGF-1 levels in each pig day 3, 6, 9 and at euthanasia in Experiment 1

| Pig ID | Litter | Treatment | Day | IGF-1 levels | Hours since last rhIGF-1 bolus |
| --- | --- | --- | --- | --- | --- |
| 701-19 | 1 | rhIGF-1 | 3 | 84.9 | 5 |
| 701-19 | 1 | rhIGF-1 | 6 | 90.2 | 3 |
| 701-19 | 1 | rhIGF-1 | 9 | 32.3 | 8 |
| 701-19 | 1 | rhIGF-1 | Euth. | 136 | 1 |
| 703-19 | 1 | rhIGF-1 | 3 | 97 | 5 |
| 703-19 | 1 | rhIGF-1 | 6 | 97.9 | 3 |
| 703-19 | 1 | rhIGF-1 | 9 | 50.6 | 8 |
| 703-19 | 1 | rhIGF-1 | Euth. | 115 | 1.8 |
| 706-19 | 1 | rhIGF-1 | 3 | 70 | 5 |
| 706-19 | 1 | rhIGF-1 | 6 | 75.5 | 3 |
| 706-19 | 1 | rhIGF-1 | 9 | 50.8 | 8 |
| 706-19 | 1 | rhIGF-1 | Euth. | 126 | 3.1 |
| 708-19 | 1 | rhIGF-1 | 3 | 59.4 | 5 |
| 708-19 | 1 | rhIGF-1 | 6 | 55.3 | 3 |
| 708-19 | 1 | rhIGF-1 | 9 | 28.9 | 8 |
| 708-19 | 1 | rhIGF-1 | Euth. | 33.6 | 3.9 |
| 700-19 | 1 | CON | 3 | 23.1 | 5 |
| 700-19 | 1 | CON | 6 | 25.8 | 3 |
| 700-19 | 1 | CON | 9 | N/A | N/A |
| 700-19 | 1 | CON | Euth. | N/A | N/A |
| 702-19 | 1 | CON | 3 | 31.1 | 5 |
| 702-19 | 1 | CON | 6 | 18.6 | 3 |
| 702-19 | 1 | CON | 9 | 20.2 | 8 |
| 702-19 | 1 | CON | Euth. | 16.6 | 1.3 |
| 704-19 | 1 | CON | 3 | 24.9 | 5 |
| 704-19 | 1 | CON | 6 | 36.4 | 3 |
| 704-19 | 1 | CON | 9 | N/A | N/A |
| 704-19 | 1 | CON | Euth. | 32.1 | 2.3 |
| 705-19 | 1 | CON | 3 | 31.7 | 5 |
| 705-19 | 1 | CON | 6 | 23.8 | 3 |
| 705-19 | 1 | CON | 9 | 29.3 | 8 |
| 705-19 | 1 | CON | Euth. | 20.8 | 2.7 |
| 707-19 | 1 | CON | 3 | 25.8 | 5 |
| 707-19 | 1 | CON | 6 | 31.6 | 3 |
| 707-19 | 1 | CON | 9 | 23.6 | 8 |
| 707-19 | 1 | CON | Euth. | 19.4 | 3.5 |
| 709-19 | 1 | CON | 3 | 34.9 | 5 |
| 709-19 | 1 | CON | 6 | 34.1 | 3 |
| 709-19 | 1 | CON | 9 | 35.8 | 8 |
| 709-19 | 1 | CON | Euth. | 26 | 4.3 |
| 711-19 | 2 | rhIGF-1 | 3 | 68.6 | 5 |
| 711-19 | 2 | rhIGF-1 | 6 | N/A | N/A |
| 711-19 | 2 | rhIGF-1 | 9 | N/A | N/A |
| 711-19 | 2 | rhIGF-1 | Euth. | N/A | N/A |
| 713-19 | 2 | rhIGF-1 | 3 | 78.8 | 5 |
| 713-19 | 2 | rhIGF-1 | 6 | 40.7 | 3 |
| 713-19 | 2 | rhIGF-1 | 9 | N/A | N/A |
| 713-19 | 2 | rhIGF-1 | Euth. | N/A | N/A |
| 718-19 | 2 | rhIGF-1 | 3 | 65.4 | 5 |
| 718-19 | 2 | rhIGF-1 | 6 | 61.7 | 3 |
| 718-19 | 2 | rhIGF-1 | 9 | 40.3 | 8 |
| 718-19 | 2 | rhIGF-1 | Euth. | 152 | 1.4 |
| 719-19 | 2 | rhIGF-1 | 3 | 90.9 | 5 |
| 719-19 | 2 | rhIGF-1 | 6 | N/A | N/A |
| 719-19 | 2 | rhIGF-1 | 9 | N/A | N/A |
| 719-19 | 2 | rhIGF-1 | Euth. | <10.0 | 1.8 |
| 720-19 | 2 | rhIGF-1 | 3 | 118 | 5 |
| 720-19 | 2 | rhIGF-1 | 6 | 83 | 3 |
| 720-19 | 2 | rhIGF-1 | 9 | N/A | N/A |
| 720-19 | 2 | rhIGF-1 | Euth. | 129 | 2.1 |
| 723-19 | 2 | rhIGF-1 | 3 | 94.2 | 5 |
| 723-19 | 2 | rhIGF-1 | 6 | 24.1 | 3 |
| 723-19 | 2 | rhIGF-1 | 9 | 65 | 8 |
| 723-19 | 2 | rhIGF-1 | Euth. | 135 | 3.1 |
| 724-19 | 2 | rhIGF-1 | 3 | 86.3 | 5 |
| 724-19 | 2 | rhIGF-1 | 6 | 98.4 | 3 |
| 724-19 | 2 | rhIGF-1 | 9 | 128 | 8 |
| 724-19 | 2 | rhIGF-1 | Euth. | 216 | 3.3 |
| 725-19 | 2 | rhIGF-1 | 3 | 90 | 5 |
| 725-19 | 2 | rhIGF-1 | 6 | 87.9 | 3 |
| 725-19 | 2 | rhIGF-1 | 9 | N/A | N/A |
| 725-19 | 2 | rhIGF-1 | Euth. | 111 | 3.8 |
| 726-19 | 2 | rhIGF-1 | 3 | 118 | 5 |
| 726-19 | 2 | rhIGF-1 | 6 | 80.8 | 3 |
| 726-19 | 2 | rhIGF-1 | 9 | N/A | N/A |
| 726-19 | 2 | rhIGF-1 | Euth. | 86.8 | 4.1 |
| 728-19 | 2 | rhIGF-1 | 3 | 65.4 | 5 |
| 728-19 | 2 | rhIGF-1 | 6 | 97.3 | 3 |
| 728-19 | 2 | rhIGF-1 | 9 | 62.3 | 8 |
| 728-19 | 2 | rhIGF-1 | Euth. | 85.5 | 5.6 |
| 729-19 | 2 | rhIGF-1 | 3 | 65.9 | 5 |
| 729-19 | 2 | rhIGF-1 | 6 | 50.8 | 3 |
| 729-19 | 2 | rhIGF-1 | 9 | 38.1 | 8 |
| 729-19 | 2 | rhIGF-1 | Euth. | 88.8 | 5.3 |
| 730-19 | 2 | rhIGF-1 | 3 | 76.7 | 5 |
| 730-19 | 2 | rhIGF-1 | 6 | N/A | N/A |
| 730-19 | 2 | rhIGF-1 | 9 | N/A | N/A |
| 730-19 | 2 | rhIGF-1 | Euth. | <10 | 6.1 |
| 732-19 | 2 | rhIGF-1 | 3 | 93 | 5 |
| 732-19 | 2 | rhIGF-1 | 6 | N/A | N/A |
| 732-19 | 2 | rhIGF-1 | 9 | N/A | N/A |
| 732-19 | 2 | rhIGF-1 | Euth. | 31.5 | 6.6 |
| 710-19 | 2 | CON | 3 | 28.1 | 5 |
| 710-19 | 2 | CON | 6 | N/A | N/A |
| 710-19 | 2 | CON | 9 | N/A | N/A |
| 710-19 | 2 | CON | Euth. | N/A | N/A |
| 712-19 | 2 | CON | 3 | 88.5 | 5 |
| 712-19 | 2 | CON | 6 | 19.6 | 3 |
| 712-19 | 2 | CON | 9 | N/A | N/A |
| 712-19 | 2 | CON | Euth. | N/A | N/A |
| 714-19 | 2 | CON | 3 | 44.1 | 5 |
| 714-19 | 2 | CON | 6 | 43.1 | 3 |
| 714-19 | 2 | CON | 9 | N/A | N/A |
| 714-19 | 2 | CON | Euth. | N/A | N/A |
| 715-19 | 2 | CON | 3 | 29 | 5 |
| 715-19 | 2 | CON | 6 | 16.7 | 3 |
| 715-19 | 2 | CON | 9 | N/A | N/A |
| 715-19 | 2 | CON | Euth. | N/A | N/A |
| 716-19 | 2 | CON | 3 | 46.5 | 5 |
| 716-19 | 2 | CON | 6 | 33.3 | 3 |
| 716-19 | 2 | CON | 9 | N/A | N/A |
| 716-19 | 2 | CON | Euth. | N/A | N/A |
| 717-19 | 2 | CON | 3 | 27.2 | 5 |
| 717-19 | 2 | CON | 6 | 13.3 | 3 |
| 717-19 | 2 | CON | 9 | 12.8 | 8 |
| 717-19 | 2 | CON | Euth. | <10 | 1 |
| 721-19 | 2 | CON | 3 | 45 | 5 |
| 721-19 | 2 | CON | 6 | 26.3 | 3 |
| 721-19 | 2 | CON | 9 | 46.7 | 8 |
| 721-19 | 2 | CON | Euth. | 38.1 | 2.4 |
| 722-19 | 2 | CON | 3 | 23.1 | 5 |
| 722-19 | 2 | CON | 6 | 16.1 | 3 |
| 722-19 | 2 | CON | 9 | N/A | N/A |
| 722-19 | 2 | CON | Euth. | 25.9 | 2.8 |
| 727-19 | 2 | CON | 3 | 47.9 | 5 |
| 727-19 | 2 | CON | 6 | 36.9 | 3 |
| 727-19 | 2 | CON | 9 | 31.4 | 8 |
| 727-19 | 2 | CON | Euth. | 33.3 | 4.9 |
| 731-19 | 2 | CON | 3 | 35.9 | 5 |
| 731-19 | 2 | CON | 6 | 11 | 3 |
| 731-19 | 2 | CON | 9 | N/A | N/A |
| 731-19 | 2 | CON | Euth. | <10 | 6.3 |
| O | 2 | CON | 3 | 42.9 | 5 |
| O | 2 | CON | 6 | N/A | N/A |
| O | 2 | CON | 9 | N/A | N/A |
| O | 2 | CON | Euth. | N/A | N/A |
| 737-19 | 3 | rhIGF-1 | 3 | 78 | 5 |
| 737-19 | 3 | rhIGF-1 | 6 | 105 | 3 |
| 737-19 | 3 | rhIGF-1 | 9 | N/A | N/A |
| 737-19 | 3 | rhIGF-1 | Euth. | N/A | N/A |
| 739-19* | 3 | rhIGF-1 | 3 | 60.6 | 5 |
| 739-19* | 3 | rhIGF-1 | 6 | 18.2 | 3 |
| 739-19* | 3 | rhIGF-1 | 9 | 13 | 8 |
| 739-19* | 3 | rhIGF-1 | Euth. | 15.4 | 1.8 |
| 740-19 | 3 | rhIGF-1 | 3 | 109 | 5 |
| 740-19 | 3 | rhIGF-1 | 6 | 59.8 | 3 |
| 740-19 | 3 | rhIGF-1 | 9 | 49.4 | 8 |
| 740-19 | 3 | rhIGF-1 | Euth. | 94.3 | 2.2 |
| 741-19 | 3 | rhIGF-1 | 3 | 86.7 | 5 |
| 741-19 | 3 | rhIGF-1 | 6 | 13.9 | 3 |
| 741-19 | 3 | rhIGF-1 | 9 | 40.9 | 8 |
| 741-19 | 3 | rhIGF-1 | Euth. | 101 | 2.5 |
| 743-19 | 3 | rhIGF-1 | 3 | 58.6 | 5 |
| 743-19 | 3 | rhIGF-1 | 6 | 33.6 | 3 |
| 743-19 | 3 | rhIGF-1 | 9 | N/A | N/A |
| 743-19 | 3 | rhIGF-1 | Euth. | N/A | N/A |
| 746-19 | 3 | rhIGF-1 | 3 | 66.2 | 5 |
| 746-19 | 3 | rhIGF-1 | 6 | 83.6 | 3 |
| 746-19 | 3 | rhIGF-1 | 9 | N/A | N/A |
| 746-19 | 3 | rhIGF-1 | Euth. | 48.5 | 4.7 |
| 747-19 | 3 | rhIGF-1 | 3 | 59.4 | 5 |
| 747-19 | 3 | rhIGF-1 | 6 | 44.4 | 3 |
| 747-19 | 3 | rhIGF-1 | 9 | 28 | 8 |
| 747-19 | 3 | rhIGF-1 | Euth. | 44.1 | 5 |
| 749-19 | 3 | rhIGF-1 | 3 | 87.2 | 5 |
| 749-19 | 3 | rhIGF-1 | 6 | N/A | N/A |
| 749-19 | 3 | rhIGF-1 | 9 | N/A | N/A |
| 749-19 | 3 | rhIGF-1 | Euth. | 76.1 | 5.7 |
| R | 3 | rhIGF-1 | 3 | 85.3 | 5 |
| R | 3 | rhIGF-1 | 6 | 48.9 | 3 |
| R | 3 | rhIGF-1 | 9 | N/A | N/A |
| R | 3 | rhIGF-1 | Euth. | N/A | N/A |
| 734-19 | 3 | CON | 3 | 30.2 | 5 |
| 734-19 | 3 | CON | 6 | 20.7 | 3 |
| 734-19 | 3 | CON | 9 | N/A | N/A |
| 734-19 | 3 | CON | Euth. | N/A | N/A |
| 735-19 | 3 | CON | 3 | 27.8 | 5 |
| 735-19 | 3 | CON | 6 | 26.9 | 3 |
| 735-19 | 3 | CON | 9 | N/A | N/A |
| 735-19 | 3 | CON | Euth. | N/A | N/A |
| 736-19 | 3 | CON | 3 | 32.2 | 5 |
| 736-19 | 3 | CON | 6 | 11.9 | 3 |
| 736-19 | 3 | CON | 9 | N/A | N/A |
| 736-19 | 3 | CON | Euth. | N/A | N/A |
| 738-19 | 3 | CON | 3 | 27.5 | 5 |
| 738-19 | 3 | CON | 6 | 29.1 | 3 |
| 738-19 | 3 | CON | 9 | 35.1 | 8 |
| 738-19 | 3 | CON | Euth. | 24.9 | 1.5 |
| 742-19 | 3 | CON | 3 | 34.2 | 5 |
| 742-19 | 3 | CON | 6 | 21.3 | 3 |
| 742-19 | 3 | CON | 9 | N/A | N/A |
| 742-19 | 3 | CON | Euth. | 14.1 | 2.8 |
| 744-19 | 3 | CON | 3 | 27.3 | 5 |
| 744-19 | 3 | CON | 6 | 29.5 | 3 |
| 744-19 | 3 | CON | 9 | 15.9 | 8 |
| 744-19 | 3 | CON | Euth. | 15.9 | 3.5 |
| 745-19 | 3 | CON | 3 | 29.3 | 5 |
| 745-19 | 3 | CON | 6 | 18.2 | 3 |
| 745-19 | 3 | CON | 9 | 12.2 | 8 |
| 745-19 | 3 | CON | Euth. | 10.7 | 3.8 |
| 748-19 | 3 | CON | 3 | 25.8 | 5 |
| 748-19 | 3 | CON | 6 | 20.5 | 3 |
| 748-19 | 3 | CON | 9 | N/A | N/A |
| 748-19 | 3 | CON | Euth. | 10.2 | 5.3 |

Blood samples day 3, 6 and 9 were taken via the umbilical catheter, blood samples at euthanasia (Euth.) day 9 were taken intracardially 1-7 h after the last subcutaneous injections of rhIGF-1 or vehicle. * Pig no. 739-19 was excluded for further analyses and sampling because of confirmed repeatedly low IGF-1 levels despite rhIGF-1 injections. rhIGF-1, recombinant human insulin-like growth factor-1; N/A, not available.

**Supplemental Table S6.** Clinical parameters of preterm pigs treated with rhIGF-1 or vehicle formulation subcutaneously for eight days in Experiment 1.

| Parameter | rhIGF-1 | Controls |
| --- | --- | --- |
| Birth weight, g | 945 ±41 | 938 ±41 |
| Temperature, 1 hr | 37.0 ±0.2 | 37.0 ±0.2 |
| Temperature, 24 hr | 38.1 ±0.2 | 38.5 ±0.2 |
| Clinical scores,  Median [Upper; Lower Limit] |  |  |
| Day 1, evening | 1 [1; 4] | 1 [1; 1] |
| Day 2  Morning, evening | 1 [1; 3]  1 [1; 2] | 1 [1; 2]  1 [1; 4] |
| Day 3  Morning, evening | 1 [1; 2]  1 [1; 1] | 1 [1; 4]  1 [1; 2] |
| Day 4  Morning, evening | 1 [1; 2]  1 [1; 1] | 1 [1; 2]  1 [1; 1] |
| Day 5  Morning, evening | 1 [1; 2]  1 [1; 2] | 1 [1; 3]  1 [1; 3] |
| Day 6  Morning, evening | 1 [1; 2]  1 [1; 2] | 1 [1; 4]  1 [1; 2] |
| Day 7  Morning, evening | 1 [1; 4]  1 [1; 1] | 1 [1; 3]  1 [1; 2] |
| Day 8  Morning, evening | 1 [1; 2]  1 [1; 1] | 1 [1; 2]  1 [1; 2] |
| Day 9, morning | 1 [1; 2] | 1 [1; 2] |
| Fecal score,  Median [Upper; Lower Limit] |  |  |
| Day 1, evening | - | - |
| Day 2  Morning, evening | 1 [1; 4]  1 [1; 4] | 1 [1; 3]  3 [1; 5] |
| Day 3  Morning, evening | 1.5 [1; 4]  3 [1; 4] | 1 [1; 3]  1 [1; 4] |
| Day 4  Morning, evening | 4 [1; 5]  4 [3; 4] | 3 [1; 5]  3 [1; 4] |
| Day 5  Morning, evening | 4 [1; 5]  3 [1; 4] | 4 [1; 4]  4 [3; 5] |
| Day 6  Morning, evening | 4 [2; 4]  4 [1; 4] | 4 [3; 5]  4 [3; 5] |
| Day 7  Morning, evening | 4 [1; 4]  3.5 [3; 4] | 4 [2; 4]  4 [2; 4] |
| Day 8  Morning, evening | 3 [2; 4]  3 [1; 4] | 3 [2; 4]  3 [1; 5] |
| Day 9, morning | 3.5 [2; 4] | 3 [2; 4] |

**Supplemental Table S7.** Clinical parameters of preterm pigs treated with rhIGF-1 or vehicle formulation intra-arterially for four days in Experiment 2

| Parameter | rhIGF-1 | Controls |
| --- | --- | --- |
| Birth weight, g | 876 ±52 | 890 ±54 |
| Temperature, 1 hr | 36.8 ±0.1 | 36.5 ±0.2 |
| Temperature, 24 hr | 37.5 ±0.1 | 37.7 ±0.1 |
| Clinical scores,  Median [Upper; Lower Limit] |  |  |
| Day 1, evening | 1 [1; 1] | 1 [1; 2] |
| Day 2  Morning, evening | 1 [1; 1]  1 [1; 1] | 1 [1; 3]  1 [1; 1] |
| Day 3  Morning, evening | 1 [1; 1]  1 [1; 1] | 1 [1; 2]  1 [1; 1] |
| Day 4  Morning, evening | 1 [1; 1]  1 [1; 2] | 1 [1; 2]  1 [1; 2] |
| Day 5, morning | 1 [1; 1] | 1 [1; 1] |
| Fecal score,  Median [Upper; Lower Limit] |  |  |
| Day 1, evening | - | - |
| Day 2  Morning, evening | 1 [1; 1]  1 [1; 3] | 1 [1; 1]  1 [1; 3] |
| Day 3  Morning, evening | 3 [3; 4]  2.5 [1; 4] | 1 [1; 3]  - |
| Day 4  Morning, evening | 3 [3; 4]  1 [1; 4] | 2 [1; 3]  3 [1; 4] |
| Day 5, morning | 3 [1; 3] | 3 [2; 4] |
